# Visualizing regulatory interactions in metabolic networks

**DOI:** 10.1186/1741-7007-5-46

**Published:** 2007-10-16

**Authors:** Stephan Noack, Aljoscha Wahl, Ermir Qeli, Wolfgang Wiechert

**Affiliations:** 1 Institute of Biotechnology 2, Research Centre Jülich, Germany; 2 MPI for Complex Technical Systems Magdeburg, Germany; 3 Department of Mathematics and Computer Science, University of Marburg, Germany; 4 Institute of Systems Engineering, Department of Simulation, University of Siegen, Germany

## Abstract

**Background:**

Direct visualization of data sets in the context of biochemical network drawings is one of the most appealing approaches in the field of data evaluation within systems biology. One important type of information that is very helpful in interpreting and understanding metabolic networks has been overlooked so far. Here we focus on the representation of this type of information given by the strength of regulatory interactions between metabolite pools and reaction steps.

**Results:**

The visualization of such interactions in a given metabolic network is based on a novel concept defining the regulatory strength (RS) of effectors regulating certain reaction steps. It is applicable to any mechanistic reaction kinetic formula. The RS values are measures for the strength of an up- or down-regulation of a reaction step compared with the completely non-inhibited or non-activated state, respectively. One numerical RS value is associated to any effector edge contained in the network. The RS is approximately interpretable on a percentage scale where 100% means the maximal possible inhibition or activation, respectively, and 0% means the absence of a regulatory interaction. If many effectors influence a certain reaction step, the respective percentages indicate the proportion in which the different effectors contribute to the total regulation of the reaction step. The benefits of the proposed method are demonstrated with a complex example system of a dynamic *E. coli *network.

**Conclusion:**

The presented visualization approach is suitable for an intuitive interpretation of simulation data of metabolic networks under dynamic as well as steady-state conditions. Huge amounts of simulation data can be analyzed in a quick and comprehensive way. An extended time-resolved graphical network presentation provides a series of information about regulatory interaction within the biological system under investigation.

## Background

Research projects in systems biology produce large amounts of data that usually spread over various 'omics' domains, are time dependent or belong to different organisms and physiological conditions. Irrespectively of whether these data are produced in a wet lab or on a computer, the evaluation requires visualization techniques representing as much information as possible in an intuitive way. Clearly, the direct visualization of data sets in the context of a biochemical network drawing is one of the most appealing approaches in this field.

This contribution is concerned with data visualization in the context of metabolic networks. It focuses on the representation of an important type of information given by the strength of regulatory interactions between metabolite pools and reaction steps. The following brief survey of visualization methods for metabolomic and fluxomic data shows that up to now metabolite pool size and flux data have been represented mainly in a network context whereas appropriate concepts to visualize regulatory information are missing.

### Visualization methods

**Figure 1:**
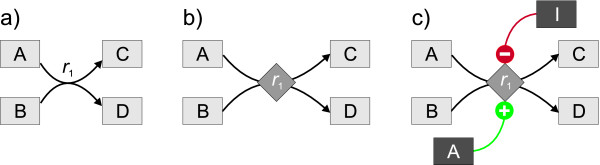
**Three possible graphs representing a small metabolic network**. (a) hypergraph, (b) bipartite graph and (c) extended bipartite graph with effector edges.

In general, a metabolic network is drawn as a directed graph where the nodes represent metabolite pools and the edges represent chemical reaction steps. As biochemical reaction steps can have multiple substrates and products, a hypergraph with multi-source multi-target edges is commonly used [1-4]. Alternatively, by introducing a second set of nodes representing the reactions, the hypergraph can be transformed into a bipartite graph with directed one-to-one edges (cf. Figure 1). This clearly has some consequences for the possible types of information visualization.

Some other conceptional differences found in the literature are whether metabolites or fluxes are allowed to be duplicated in order to avoid edge intersections or whether cometabolites are distinguished optically from reaction substrates and products. In any case, the data to be visualized can be linked directly to the nodes or edges of the network. This can basically be achieved in the following ways.

**Figure 2:**
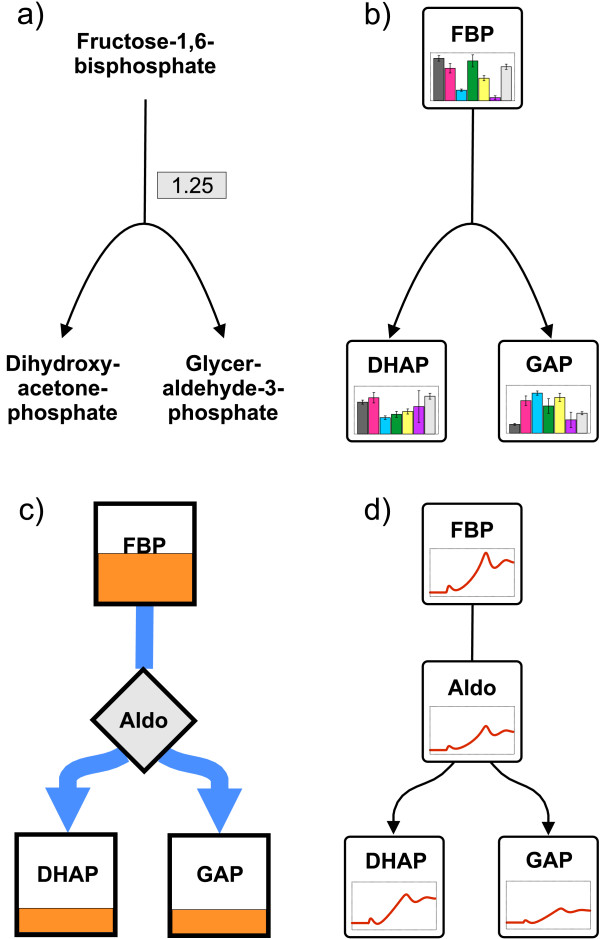
**Different ways to represent pool size and flux data in the context of a metabolic network**. (a) Network nodes and edges of a hypergraph are annotated with textual tags. (b) Direct representation of metabolite data in a hypergraph using bar plots. (c) Mapping of pool sizes and flux quantities to the shape and size of the network nodes using a bipartite graph. Metabolite concentrations are represented as boxes filled depending on their pool size. Fluxes are represented by varying arrow widths. (d) Visualization of time-dependent data in a bipartite graph using time course plots.

• The most primitive way to represent data in the network context is to annotate the nodes or edges with textual tags (cf. Figure 2a). Although this is not really a graphical representation, it has the big advantage of being precise and offers the possibility of representing non-quantitative information along with the network [5,6].

• A direct representation of metabolite or flux data is given by mapping numerical values to the size, color or shape of the drawn network nodes [7,8]. For example, pool sizes are frequently visualized by bar plots, level meters or the size of the respective pool symbol (cf. Figure 2b,c). If fluxes are modeled as separate nodes in a bipartite graph, the same visualization options are available.

• Similarly, if fluxes are represented by hyperedges, the width of an arrow can be modified to represent the flux quantity (cf. Figure 2c).

• The situation becomes more difficult when dynamic (i.e. time-dependent) data have to be displayed. One option then is to show time course plots along with the network nodes or edges (cf. Figure 2d). Another idea is to use dynamic visualization features by producing videos with changing pool size and flux data over time. Taking snapshots from this video can be interactively facilitated by using a slider [8].

• Another frequent task is the visual comparison of different data sets that are related to different physiological conditions, different organisms, experiments versus simulations or dynamic system states at different times. In this situation the direct representation by changing the appearance of nodes or edges is still applicable. Typically, this results in a bar chart replacing or annotating the network nodes [9].

• Another option is the representation of multiple copies of the whole network. As a special case, 2.5D representations for data comparison have been developed by stacking network plots in three dimensions [10,11].

• Finally, the comparison of different time plots along with the symbols is even capable of comparing several complete time courses, although this approach becomes difficult to percept with a growing number of curves.

### Regulatory information

All of these methods are well established and implemented in various software tools for network-based visualization [5-11]. However, there is still one important type of information missing that is very helpful for interpreting and understanding the function of metabolic networks. It is related to the strength of regulatory interactions between metabolite pools and the reaction steps influenced by these pools. Biologists are used to including graphical representations of these interactions by drawing interaction edges connecting pools and fluxes. These edges are usually labeled with a plus or minus sign for activating or inhibiting interactions, respectively. Clearly, this representation is only possible when fluxes are explicitly displayed as nodes in a bipartite graph.

Interestingly, this qualitative regulatory information has never been represented in a quantitative way in the available visualization tools. This would be a valuable complement to the already displayed pool size and flux data. If, for example, a flux is down-regulated although its substrate pools are at high levels and product pools are at low levels, the cause must be an inhibitory effect of some other metabolite pool. Thus, the incorporation of additional edges for inhibitors and activators would help to explain why metabolic fluxes are at their present levels.

The major problem here is obtaining a precise definition of what is meant by the 'regulatory strength' (RS) of an interaction. The goal of this contribution is to develop such a definition which is suitable for the intuitive interpretation of data under dynamic and steady-state conditions. Clearly, such a definition can only be reasonably given for the case of simulated data because some information on the reaction kinetics of the involved steps is needed to establish a meaningful RS definition.

This contribution is organized as follows. A novel concept for the determination of the RS of effectors in enzyme-catalyzed reactions is presented in the first two sections. Next, a general definition for the RS is given followed by a description of the visualization approach. Finally, we provide an example to demonstrate the practical significance of the proposed method, where the whole concept is applied to a relevant dynamic model system of *E. coli*.

### The concept of RS

#### Properties of RS

Before explaining in detail how the RS for metabolite pools influencing reaction steps is defined, a list of properties is given that should be reasonably fulfilled by the new concept. The driving force behind these properties is to ensure a maximum of intuitive interpretability and to avoid an overload of information for the user.

(i) A RS is defined for all effectors (i.e. inhibitors or activators) of a reaction step which are not contained in the set of substrates or products. These effectors can be identified immediately from the corresponding reaction kinetic expression.

(ii) One numerical RS value should be associated to any effector edge contained in the network. Thus, it is possible to visualize RSs directly in the network context. Any of the already-mentioned visualization techniques for pool sizes and fluxes might be used for this purpose.

(iii) The RS of an effector with respect to a reaction step has to be calculated from the momentary values of pool sizes and fluxes in the network with the additional knowledge of the respective reaction kinetic formula and parameters. Consequently, RS is a time-dependent quantity which does not depend on the history of a current system state.

(iv) The RS should express how strong an influence a reaction step has on a given reaction rate. Moreover, it should distinguish between activation relations (positive sign) and inhibition relations (negative sign). In the visualization the cases can be distinguished between easily by using different colors.

(v) The RS should be approximately interpretable on a percentage scale where 100% means the maximal possible inhibition or activation and 0% means the absence of a regulatory interaction.

(vi) If many effectors have an influence on a certain reaction step, the respective percentages should indicate the proportion in which the different effectors contribute to the total regulation of the reaction step.

Here some comments concerning the reasoning and rationale behind these properties are appropriate. Referring to item (i), the definition of RSs for reaction substrates and products (reversible reaction only) is, in principle, possible. However, the obtained values would not indicate any metabolic regulation, but rather how strong the reaction is driven by the availability of substrates and products, respectively. This information can be directly represented by the visualization of metabolic pool sizes and fluxes. In most cases the effectors of an enzymatic reaction are not consumed by the reaction step itself. The only exceptions are substrate and product inhibition mechanisms which are explicitly denoted in the reaction kinetic formula and would also, therefore, be covered by the RS definition.

It is reasonable to quantitate the effector influence by exactly one RS value, otherwise the multitude of visualized information is likely to become confusing (item (ii)). Moreover, for the RS calculation, the general assumption is made that a certain effector molecule modulating an enzymatic reaction step is instantaneously available and distributed equally over the whole cell (item (iii)).

The properties in items (iv)–(vi) are important for a meaningful and intuitive interpretation of RSs. In particular, the distinction between activators and inhibitors is of fundamental importance with respect to the underlying effect of a metabolic enzyme regulation. With regards to the practical implementation of RS values, the definitions of lower and upper bounds are indispensable. In addition, applying a percentage scale facilitates the reception of information.

#### Conceptual problems in the definition of RS

When trying to construct a RS measure that fulfills these conditions it became clear that different approaches are possible and some decisions have to be made. Moreover, it turns out in the following that the above-mentioned requirements are not completely free of contradictions so that some compromises are necessary. However, it is important to note that the precise value of a displayed quantity plays no role in a graphical visualization, but, rather, it is the rough order of magnitude that is important. Thus, contradictions are not important if they can be resolved by sacrificing some numerical precision.

One conceptional difficulty with the introduction of a RS is that the activation or inhibition state of a reaction step in relation to the state where all activators or inhibitors are absent must be quantitated by exactly *k *values, where *k *is the number of effectors. This immediately indicates the implicit assumption that activators and inhibitors act independently in a reaction. In contrast, it is well known from enzyme kinetics that this is not always the case. However, if correlations between the influences of different effectors have to be taken into account, further coefficients of higher order are needed to characterize this correlation. Clearly, this would prevent us from implementing an intuitive network-based visualization.

A well-known family of methods that assigns exactly one coefficient to each effector are the sensitivity-based methods from which the elasticities defined in metabolic control theory are the best-known example. Although elasticities play an important role in metabolic control theory, they are certainly not the right quantities to be used for visualization in the way specified above. This can be explained easily with an inhibitory relationship expressed by a multiplicative hyperbolic term in a reaction kinetic expression (here S is substrate concentration and I is inhibitor concentration):

r(S,I)=rmax⁡SKS(1+I/KI)+S
 MathType@MTEF@5@5@+=feaafiart1ev1aaatCvAUfKttLearuWrP9MDH5MBPbIqV92AaeXatLxBI9gBaebbnrfifHhDYfgasaacH8akY=wiFfYdH8Gipec8Eeeu0xXdbba9frFj0=OqFfea0dXdd9vqai=hGuQ8kuc9pgc9s8qqaq=dirpe0xb9q8qiLsFr0=vr0=vr0dc8meaabaqaciaacaGaaeqabaqabeGadaaakeaacqWGYbGCcqGGOaakcqqGtbWucqGGSaalcqqGjbqscqGGPaqkcqGH9aqpdaWcaaqaaiabdkhaYnaaBaaaleaacyGGTbqBcqGGHbqycqGG4baEaeqaaOGaee4uamfabaGaem4saS0aaSbaaSqaaiabbofatbqabaGccqGGOaakcqaIXaqmcqGHRaWkcqqGjbqscqGGVaWlcqWGlbWsdaWgaaWcbaGaeeysaKeabeaakiabcMcaPiabgUcaRiabbofatbaaaaa@477F@

In this example the sensitivity ∂*r*/∂I tends to zero with increasing inhibitor concentration which would erroneously indicate that the inhibitor has no effect on reaction flux. Obviously, the opposite is true and RS should tend to -100% in this case. Consequently, when used for network visualization, elasticities rather produce non-intuitive results.

Likewise, the use of flux control coefficients is not appropriate because these scaled sensitivities reflect the global network regulation (i.e. the joint action of all reaction steps) and thus cannot be interpreted locally for one isolated reaction step in the network.

In this contribution the aim is now to find a quantity expressing how strong a reaction step is up- or down-regulated compared with the completely non-inhibited or non-activated state, respectively. As a first example, in Equation 1 the RS can be reasonable defined by

νI=r(S,I)−r(S,I=0)r(S,I=0)−r(S,I→∞)=KS+SKS(1+I/KI)+S−1
 MathType@MTEF@5@5@+=feaafiart1ev1aaatCvAUfKttLearuWrP9MDH5MBPbIqV92AaeXatLxBI9gBaebbnrfifHhDYfgasaacH8akY=wiFfYdH8Gipec8Eeeu0xXdbba9frFj0=OqFfea0dXdd9vqai=hGuQ8kuc9pgc9s8qqaq=dirpe0xb9q8qiLsFr0=vr0=vr0dc8meaabaqaciaacaGaaeqabaqabeGadaaakeaafaqaaeGadaaabaacciGae8xVd42aaSbaaSqaaGqaaiab+LeajbqabaaakeaacqGH9aqpaeaadaWcaaqaaiabdkhaYjabcIcaOiabbofatjabcYcaSiabbMeajjabcMcaPiabgkHiTiabdkhaYjabcIcaOiabbofatjabcYcaSiabbMeajjabg2da9iabicdaWiabcMcaPaqaaiabdkhaYjabcIcaOiabbofatjabcYcaSiabbMeajjabg2da9iabicdaWiabcMcaPiabgkHiTiabdkhaYjabcIcaOiabbofatjabcYcaSiabbMeajjabgkziUkabg6HiLkabcMcaPaaaaeaaaeaacqGH9aqpaeaadaWcaaqaaiabdUealnaaBaaaleaacqqGtbWuaeqaaOGaey4kaSIaee4uamfabaGaem4saS0aaSbaaSqaaiabbofatbqabaGccqGGOaakcqaIXaqmcqGHRaWkcqqGjbqscqGGVaWlcqWGlbWsdaWgaaWcbaGaeeysaKeabeaakiabcMcaPiabgUcaRiabbofatbaacqGHsislcqaIXaqmaaaaaa@6722@

because this is the percentage by which the non-inhibited flux (I = 0) is down-regulated.

In some cases enzymatic reactions are described by kinetic expressions that show no saturation behavior, i.e. the flux continuously increases with increasing activator concentration. As an example for a mechanistic enzyme description with these properties the following kinetic expression is given, describing an allosterically activated enzyme covering *n *binding sites for an activator:

r(S,A)=rmax⁡S(1+(A/KA)n)KS+S
 MathType@MTEF@5@5@+=feaafiart1ev1aaatCvAUfKttLearuWrP9MDH5MBPbIqV92AaeXatLxBI9gBaebbnrfifHhDYfgasaacH8akY=wiFfYdH8Gipec8Eeeu0xXdbba9frFj0=OqFfea0dXdd9vqai=hGuQ8kuc9pgc9s8qqaq=dirpe0xb9q8qiLsFr0=vr0=vr0dc8meaabaqaciaacaGaaeqabaqabeGadaaakeaacqWGYbGCcqGGOaakcqqGtbWucqGGSaalcqqGbbqqcqGGPaqkcqGH9aqpdaWcaaqaaiabdkhaYnaaBaaaleaacyGGTbqBcqGGHbqycqGG4baEaeqaaOGaee4uamLaeiikaGIaeGymaeJaey4kaSIaeiikaGIaeeyqaeKaei4la8Iaem4saS0aaSbaaSqaaiabbgeabbqabaGccqGGPaqkdaahaaWcbeqaaiabd6gaUbaakiabcMcaPaqaaiabdUealnaaBaaaleaacqqGtbWuaeqaaOGaey4kaSIaee4uamfaaaaa@4A9D@

The determination of the RS for the activator A using the formula

νA=r(S,A)−r(S,A=0)r(S,A→∞)−r(S,A=0)
 MathType@MTEF@5@5@+=feaafiart1ev1aaatCvAUfKttLearuWrP9MDH5MBPbIqV92AaeXatLxBI9gBaebbnrfifHhDYfgasaacH8akY=wiFfYdH8Gipec8Eeeu0xXdbba9frFj0=OqFfea0dXdd9vqai=hGuQ8kuc9pgc9s8qqaq=dirpe0xb9q8qiLsFr0=vr0=vr0dc8meaabaqaciaacaGaaeqabaqabeGadaaakeaaiiGacqWF9oGBdaWgaaWcbaGaeeyqaeeabeaakiabg2da9maalaaabaGaemOCaiNaeiikaGIaee4uamLaeiilaWIaeeyqaeKaeiykaKIaeyOeI0IaemOCaiNaeiikaGIaee4uamLaeiilaWIaeeyqaeKaeyypa0JaeGimaaJaeiykaKcabaGaemOCaiNaeiikaGIaee4uamLaeiilaWIaeeyqaeKaeyOKH4QaeyOhIuQaeiykaKIaeyOeI0IaemOCaiNaeiikaGIaee4uamLaeiilaWIaeeyqaeKaeyypa0JaeGimaaJaeiykaKcaaaaa@52B4@

will not succeed, because the limit calculation for arbitrary high activator concentrations (A → ∞) leads to infinitely high reaction rates *r*(S, A). Consequently, this results in a value of zero for *ν*_A_. For this reason the definition of an upper bound A_max _for the activator concentration is suitable. This boundary should be chosen according to the expected physiological concentration range of the respective effector metabolite.

Moreover, the corresponding simulated values must also be restricted to this range. In order to derive a general definition for the RS, upper bounds **e**_max _for all effectors are defined. However, in the case of kinetics with saturation behavior, the maximum effector concentrations need not be limited to a finite value for RS calculability.

It turns out that for arbitrary reaction kinetic formulae the definition of a RS is not as simple and straightforward as in the example from Equation 1. For this reason, the concept of RS is defined in the following in a step-by-step approach that starts with simple standard reaction kinetic formulae and successively generalizes the introduced concepts to the most general case. At the end it will be possible to apply the concept to any mechanistic reaction kinetics.

### Derivation of a general RS definition

#### Example system

**Figure 3:**
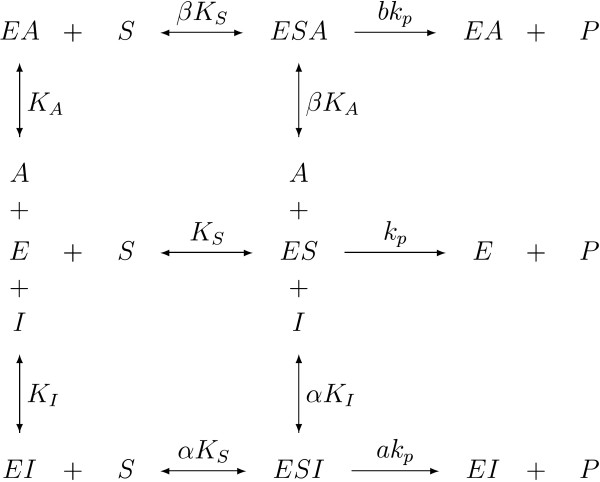
**Enzymatic reaction system with one substrate and two effectors**. The substrate S, the inhibitor I and the activator A bind to the enzyme at different sites to yield ES, EI, ESI, EA and ESA complexes. Binding of the inhibitor reduces the affinity of the substrate for the enzyme and/or the rate *k*_p_ at which product is formed. The activator has the same, but opposite, effect (i.e. the affinity and/or *k*_p _increase).

As an instructive example, consider an enzymatic reaction where the conversion of one substrate is regulated by two effectors (cf. Figure 3). Some quasi-stationarity assumptions are used for simplicity.

In this system, the inhibitor and the activator are competitive with respect to each other, i.e. the binding of one excludes the binding of the other. The velocity equation for this system in Michaelis-Menten form is [12]

r(S,A,I)=rmax⁡S[KS((1+IKI+AKA)(1+aIαKI+bAβKA)−1)+S((1+IαKI+AβKA)(1+aIαKI+bAβKA)−1)]−1
 MathType@MTEF@5@5@+=feaafiart1ev1aaatCvAUfKttLearuWrP9MDH5MBPbIqV92AaeXatLxBI9gBaebbnrfifHhDYfgasaacH8akY=wiFfYdH8Gipec8Eeeu0xXdbba9frFj0=OqFfea0dXdd9vqai=hGuQ8kuc9pgc9s8qqaq=dirpe0xb9q8qiLsFr0=vr0=vr0dc8meaabaqaciaacaGaaeqabaqabeGadaaakeaafaqadeGabaaabaGaemOCaiNaeiikaGIaee4uamLaeiilaWIaeeyqaeKaeiilaWIaeeysaKKaeiykaKIaeyypa0JaemOCai3aaSbaaSqaaiGbc2gaTjabcggaHjabcIha4bqabaGccqqGtbWudaWabaqaaiabdUealnaaBaaaleaacqqGtbWuaeqaaOWaaeWaaeaadaqadaqaaiabigdaXiabgUcaRmaalaaabaGaeeysaKeabaGaem4saS0aaSbaaSqaaiabbMeajbqabaaaaOGaey4kaSYaaSaaaeaacqqGbbqqaeaacqWGlbWsdaWgaaWcbaGaeeyqaeeabeaaaaaakiaawIcacaGLPaaadaqadaqaaiabigdaXiabgUcaRmaalaaabaGaemyyaeMaeeysaKeabaacciGae8xSdeMaem4saS0aaSbaaSqaaiabbMeajbqabaaaaOGaey4kaSYaaSaaaeaacqWGIbGycqqGbbqqaeaacqWFYoGycqWGlbWsdaWgaaWcbaGaeeyqaeeabeaaaaaakiaawIcacaGLPaaadaahaaWcbeqaaiabgkHiTiabigdaXaaaaOGaayjkaiaawMcaaaGaay5waaaabaWaamGaaeaacqGHRaWkcqqGtbWudaqadaqaamaabmaabaGaeGymaeJaey4kaSYaaSaaaeaacqqGjbqsaeaacqWFXoqycqWGlbWsdaWgaaWcbaGaeeysaKeabeaaaaGccqGHRaWkdaWcaaqaaiabbgeabbqaaiab=j7aIjabdUealnaaBaaaleaacqqGbbqqaeqaaaaaaOGaayjkaiaawMcaamaabmaabaGaeGymaeJaey4kaSYaaSaaaeaacqWGHbqycqqGjbqsaeaacqWFXoqycqWGlbWsdaWgaaWcbaGaeeysaKeabeaaaaGccqGHRaWkdaWcaaqaaiabdkgaIjabbgeabbqaaiab=j7aIjabdUealnaaBaaaleaacqqGbbqqaeqaaaaaaOGaayjkaiaawMcaamaaCaaaleqabaGaeyOeI0IaeGymaedaaaGccaGLOaGaayzkaaaacaGLDbaadaahaaWcbeqaaiabgkHiTiabigdaXaaaaaaaaa@88BD@

This example system is used in the following to derive a general RS definition including different kinetic types.

#### Enzyme kinetics with one effector

First, consider an enzyme that only possesses binding sites for the substrate and one inhibitor. Regarding the reaction scheme given above, such a system can be described by neglecting the activator influence (A = 0). The velocity equation is then given by

r(S,I)=rmax⁡S[KS((1+IKI)(1+aIαKI)−1)+S((1+IαKI)(1+aIαKI)−1)]−1,a≥0, α>0
 MathType@MTEF@5@5@+=feaafiart1ev1aaatCvAUfKttLearuWrP9MDH5MBPbIqV92AaeXatLxBI9gBaebbnrfifHhDYfgasaacH8akY=wiFfYdH8Gipec8Eeeu0xXdbba9frFj0=OqFfea0dXdd9vqai=hGuQ8kuc9pgc9s8qqaq=dirpe0xb9q8qiLsFr0=vr0=vr0dc8meaabaqaciaacaGaaeqabaqabeGadaaakeaafaqabeqacaaabaGaemOCaiNaeiikaGIaee4uamLaeiilaWIaeeysaKKaeiykaKIaeyypa0JaemOCai3aaSbaaSqaaiGbc2gaTjabcggaHjabcIha4bqabaGccqqGtbWudaWadaqaaiabdUealnaaBaaaleaacqqGtbWuaeqaaOWaaeWaaeaadaqadaqaaiabigdaXiabgUcaRmaalaaabaGaeeysaKeabaGaem4saS0aaSbaaSqaaiabbMeajbqabaaaaaGccaGLOaGaayzkaaWaaeWaaeaacqaIXaqmcqGHRaWkdaWcaaqaaiabdggaHjabbMeajbqaaGGaciab=f7aHjabdUealnaaBaaaleaacqqGjbqsaeqaaaaaaOGaayjkaiaawMcaamaaCaaaleqabaGaeyOeI0IaeGymaedaaaGccaGLOaGaayzkaaGaey4kaSIaee4uam1aaeWaaeaadaqadaqaaiabigdaXiabgUcaRmaalaaabaGaeeysaKeabaGae8xSdeMaem4saS0aaSbaaSqaaiabbMeajbqabaaaaaGccaGLOaGaayzkaaWaaeWaaeaacqaIXaqmcqGHRaWkdaWcaaqaaiabdggaHjabbMeajbqaaiab=f7aHjabdUealnaaBaaaleaacqqGjbqsaeqaaaaaaOGaayjkaiaawMcaamaaCaaaleqabaGaeyOeI0IaeGymaedaaaGccaGLOaGaayzkaaaacaGLBbGaayzxaaWaaWbaaSqabeaacqGHsislcqaIXaqmaaGccqGGSaalaeaacqWGHbqycqGHLjYScqaIWaamcqGGSaalcqqGGaaicqWFXoqycqGH+aGpcqaIWaamaaaaaa@7819@

The most common inhibition mechanisms can be derived from this equation, i.e. competitive (*α *→ ∞), non-competitive (*a *= 0, *α *= 1) and partial competitive (*a *< 1, *α *> 1) inhibition [12].

In general the influence of inhibitor I on flux *r *can be quantified as

νI=r(S,I)−rmax⁡,I(S)rmax⁡,I(S)−rmin⁡,I(S)
 MathType@MTEF@5@5@+=feaafiart1ev1aaatCvAUfKttLearuWrP9MDH5MBPbIqV92AaeXatLxBI9gBaebbnrfifHhDYfgasaacH8akY=wiFfYdH8Gipec8Eeeu0xXdbba9frFj0=OqFfea0dXdd9vqai=hGuQ8kuc9pgc9s8qqaq=dirpe0xb9q8qiLsFr0=vr0=vr0dc8meaabaqaciaacaGaaeqabaqabeGadaaakeaaiiGacqWF9oGBdaWgaaWcbaGaeeysaKeabeaakiabg2da9maalaaabaGaemOCaiNaeiikaGIaee4uamLaeiilaWIaeeysaKKaeiykaKIaeyOeI0IaemOCai3aaSbaaSqaaiGbc2gaTjabcggaHjabcIha4jabcYcaSiabbMeajbqabaGccqGGOaakcqqGtbWucqGGPaqkaeaacqWGYbGCdaWgaaWcbaGagiyBa0MaeiyyaeMaeiiEaGNaeiilaWIaeeysaKeabeaakiabcIcaOiabbofatjabcMcaPiabgkHiTiabdkhaYnaaBaaaleaacyGGTbqBcqGGPbqAcqGGUbGBcqGGSaalcqqGjbqsaeqaaOGaeiikaGIaee4uamLaeiykaKcaaaaa@58CE@

with

rmax⁡,I(S)=max⁡0≤I≤Imax⁡r(S,I)
 MathType@MTEF@5@5@+=feaafiart1ev1aaatCvAUfKttLearuWrP9MDH5MBPbIqV92AaeXatLxBI9gBaebbnrfifHhDYfgasaacH8akY=wiFfYdH8Gipec8Eeeu0xXdbba9frFj0=OqFfea0dXdd9vqai=hGuQ8kuc9pgc9s8qqaq=dirpe0xb9q8qiLsFr0=vr0=vr0dc8meaabaqaciaacaGaaeqabaqabeGadaaakeaacqWGYbGCdaWgaaWcbaGagiyBa0MaeiyyaeMaeiiEaGNaeiilaWIaeeysaKeabeaakiabcIcaOiabbofatjabcMcaPiabg2da9maaxababaGagiyBa0MaeiyyaeMaeiiEaGhaleaacqaIWaamcqGHKjYOcqqGjbqscqGHKjYOcqqGjbqsdaWgaaadbaGagiyBa0MaeiyyaeMaeiiEaGhabeaaaSqabaGccqWGYbGCcqGGOaakcqqGtbWucqGGSaalcqqGjbqscqGGPaqkaaa@4DE9@

rmin⁡,I(S)=min⁡0≤I≤Imax⁡r(S,I)
 MathType@MTEF@5@5@+=feaafiart1ev1aaatCvAUfKttLearuWrP9MDH5MBPbIqV92AaeXatLxBI9gBaebbnrfifHhDYfgasaacH8akY=wiFfYdH8Gipec8Eeeu0xXdbba9frFj0=OqFfea0dXdd9vqai=hGuQ8kuc9pgc9s8qqaq=dirpe0xb9q8qiLsFr0=vr0=vr0dc8meaabaqaciaacaGaaeqabaqabeGadaaakeaacqWGYbGCdaWgaaWcbaGagiyBa0MaeiyAaKMaeiOBa4MaeiilaWIaeeysaKeabeaakiabcIcaOiabbofatjabcMcaPiabg2da9maaxababaGagiyBa0MaeiyAaKMaeiOBa4galeaacqaIWaamcqGHKjYOcqqGjbqscqGHKjYOcqqGjbqsdaWgaaadbaGagiyBa0MaeiyyaeMaeiiEaGhabeaaaSqabaGccqWGYbGCcqGGOaakcqqGtbWucqGGSaalcqqGjbqscqGGPaqkaaa@4DE1@

The term *r*_max, I_(S) denotes the reaction rate for fixed substrate concentration S and a negligible influence of the inhibitor (I → 0). In contrast, *r*_min, I_(S) is calculated by assuming a high concentration for the inhibitor (I → I_max_). For a small inhibition the present flux *r*(S, I) is close to the maximum occurring flux *r*_max, I_(S), i.e. *ν*_I _tends to zero. Conversely, for a strong inhibition *r*(S, I) is near the minimum flux *r*_min, I_(S) and *ν*_I _tends to -100%. In the case of a partially competitive inhibition, *r*_min, I_(S) > 0 holds. Hence, the present flux is scaled between the maximum and minimum inhibitor influence.

The same approach can also be applied to a kinetic expression describing the action of an enzyme possessing only binding sites for the substrate and one activator. The equilibria follow from Figure 3 by neglecting the inhibitor (I = 0). In analogy to Equation 6, the velocity equation is given by

r(S,A)=rmax⁡S[KS((1+AKA)(1+bAβKA)−1)+S((1+AβKA)(1+bAβKA)−1)]−1,b≥0, β>0
 MathType@MTEF@5@5@+=feaafiart1ev1aaatCvAUfKttLearuWrP9MDH5MBPbIqV92AaeXatLxBI9gBaebbnrfifHhDYfgasaacH8akY=wiFfYdH8Gipec8Eeeu0xXdbba9frFj0=OqFfea0dXdd9vqai=hGuQ8kuc9pgc9s8qqaq=dirpe0xb9q8qiLsFr0=vr0=vr0dc8meaabaqaciaacaGaaeqabaqabeGadaaakeaafaqabeqacaaabaGaemOCaiNaeiikaGIaee4uamLaeiilaWIaeeyqaeKaeiykaKIaeyypa0JaemOCai3aaSbaaSqaaiGbc2gaTjabcggaHjabcIha4bqabaGccqqGtbWudaWadaqaaiabdUealnaaBaaaleaacqqGtbWuaeqaaOWaaeWaaeaadaqadaqaaiabigdaXiabgUcaRmaalaaabaGaeeyqaeeabaGaem4saS0aaSbaaSqaaiabbgeabbqabaaaaaGccaGLOaGaayzkaaWaaeWaaeaacqaIXaqmcqGHRaWkdaWcaaqaaiabdkgaIjabbgeabbqaaGGaciab=j7aIjabdUealnaaBaaaleaacqqGbbqqaeqaaaaaaOGaayjkaiaawMcaamaaCaaaleqabaGaeyOeI0IaeGymaedaaaGccaGLOaGaayzkaaGaey4kaSIaee4uam1aaeWaaeaadaqadaqaaiabigdaXiabgUcaRmaalaaabaGaeeyqaeeabaGae8NSdiMaem4saS0aaSbaaSqaaiabbgeabbqabaaaaaGccaGLOaGaayzkaaWaaeWaaeaacqaIXaqmcqGHRaWkdaWcaaqaaiabdkgaIjabbgeabbqaaiab=j7aIjabdUealnaaBaaaleaacqqGbbqqaeqaaaaaaOGaayjkaiaawMcaamaaCaaaleqabaGaeyOeI0IaeGymaedaaaGccaGLOaGaayzkaaaacaGLBbGaayzxaaWaaWbaaSqabeaacqGHsislcqaIXaqmaaGccqGGSaalaeaacqWGIbGycqGHLjYScqaIWaamcqGGSaalcqqGGaaicqWFYoGycqGH+aGpcqaIWaamaaaaaa@7797@

For a purely activating influence of A, *b *≥ 1, *β *< 1 must hold. The RS of the activator A is then defined as

νA=r(S,A)−rmin⁡,A(S)rmax⁡,A(S)−rmin⁡,A(S)
 MathType@MTEF@5@5@+=feaafiart1ev1aaatCvAUfKttLearuWrP9MDH5MBPbIqV92AaeXatLxBI9gBaebbnrfifHhDYfgasaacH8akY=wiFfYdH8Gipec8Eeeu0xXdbba9frFj0=OqFfea0dXdd9vqai=hGuQ8kuc9pgc9s8qqaq=dirpe0xb9q8qiLsFr0=vr0=vr0dc8meaabaqaciaacaGaaeqabaqabeGadaaakeaaiiGacqWF9oGBdaWgaaWcbaGaeeyqaeeabeaakiabg2da9maalaaabaGaemOCaiNaeiikaGIaee4uamLaeiilaWIaeeyqaeKaeiykaKIaeyOeI0IaemOCai3aaSbaaSqaaiGbc2gaTjabcMgaPjabc6gaUjabcYcaSiabbgeabbqabaGccqGGOaakcqqGtbWucqGGPaqkaeaacqWGYbGCdaWgaaWcbaGagiyBa0MaeiyyaeMaeiiEaGNaeiilaWIaeeyqaeeabeaakiabcIcaOiabbofatjabcMcaPiabgkHiTiabdkhaYnaaBaaaleaacyGGTbqBcqGGPbqAcqGGUbGBcqGGSaalcqqGbbqqaeqaaOGaeiikaGIaee4uamLaeiykaKcaaaaa@587A@

with

rmax⁡,A(S)=max⁡0≤A≤Amax⁡r(S,A)
 MathType@MTEF@5@5@+=feaafiart1ev1aaatCvAUfKttLearuWrP9MDH5MBPbIqV92AaeXatLxBI9gBaebbnrfifHhDYfgasaacH8akY=wiFfYdH8Gipec8Eeeu0xXdbba9frFj0=OqFfea0dXdd9vqai=hGuQ8kuc9pgc9s8qqaq=dirpe0xb9q8qiLsFr0=vr0=vr0dc8meaabaqaciaacaGaaeqabaqabeGadaaakeaacqWGYbGCdaWgaaWcbaGagiyBa0MaeiyyaeMaeiiEaGNaeiilaWIaeeyqaeeabeaakiabcIcaOiabbofatjabcMcaPiabg2da9maaxababaGagiyBa0MaeiyyaeMaeiiEaGhaleaacqaIWaamcqGHKjYOcqqGbbqqcqGHKjYOcqqGbbqqdaWgaaadbaGagiyBa0MaeiyyaeMaeiiEaGhabeaaaSqabaGccqWGYbGCcqGGOaakcqqGtbWucqGGSaalcqqGbbqqcqGGPaqkaaa@4DA9@

rmin⁡,A(S)=min⁡0≤A≤Amax⁡r(S,A)
 MathType@MTEF@5@5@+=feaafiart1ev1aaatCvAUfKttLearuWrP9MDH5MBPbIqV92AaeXatLxBI9gBaebbnrfifHhDYfgasaacH8akY=wiFfYdH8Gipec8Eeeu0xXdbba9frFj0=OqFfea0dXdd9vqai=hGuQ8kuc9pgc9s8qqaq=dirpe0xb9q8qiLsFr0=vr0=vr0dc8meaabaqaciaacaGaaeqabaqabeGadaaakeaacqWGYbGCdaWgaaWcbaGagiyBa0MaeiyAaKMaeiOBa4MaeiilaWIaeeyqaeeabeaakiabcIcaOiabbofatjabcMcaPiabg2da9maaxababaGagiyBa0MaeiyAaKMaeiOBa4galeaacqaIWaamcqGHKjYOcqqGbbqqcqGHKjYOcqqGbbqqdaWgaaadbaGagiyBa0MaeiyyaeMaeiiEaGhabeaaaSqabaGccqWGYbGCcqGGOaakcqqGtbWucqGGSaalcqqGbbqqcqGGPaqkaaa@4DA1@

For a strong activation, the flux *r*(S, A) is close to *r*_max, A_(S) and, therefore, *ν*_A _tends to 100%. Vice versa, if there is only a small activation, *r*(S, A) is in the range of *r*_min, A_(S) and *ν*_A _is near zero. Clearly, depending on the sign of the RS value a distinction between inhibiting or activating influences of the effector can be made.

#### Multiple effectors of equal directed influence

Many enzymatic reactions exist within the different metabolic pathways. The activities of these reactions are regulated by simultaneously operating effectors. As a simple example, consider an enzyme with binding sites for substrate S and two activators A_1 _and A_2_. The system is identical to that of Equation 5 if we substitute A and I by A_1 _and A_2_, respectively. In addition, the system is subjected to the restrictions that *α*, *β *< 1 (partial competitive activation), *a*, *b *> 1 (partial non-competitive activation) or both (mixed-type activation) [12].

To quantify the combined effect of both activators, the same approach as described above is chosen, i.e. the present reaction rate *r*(S, A_1_, A_2_) is put into a relation to the completely activated and non-activated state, respectively:

νres=r(S,A1,A2)−rmin⁡,A(S)rmax⁡,A(S)−rmin⁡,A(S)
 MathType@MTEF@5@5@+=feaafiart1ev1aaatCvAUfKttLearuWrP9MDH5MBPbIqV92AaeXatLxBI9gBaebbnrfifHhDYfgasaacH8akY=wiFfYdH8Gipec8Eeeu0xXdbba9frFj0=OqFfea0dXdd9vqai=hGuQ8kuc9pgc9s8qqaq=dirpe0xb9q8qiLsFr0=vr0=vr0dc8meaabaqaciaacaGaaeqabaqabeGadaaakeaaiiGacqWF9oGBdaWgaaWcbaGaeeOCaiNaeeyzauMaee4Camhabeaakiabg2da9maalaaabaGaemOCaiNaeiikaGIaee4uamLaeiilaWIaeeyqae0aaSbaaSqaaiabbgdaXaqabaGccqGGSaalcqqGbbqqdaWgaaWcbaGaeGOmaidabeaakiabcMcaPiabgkHiTiabdkhaYnaaBaaaleaacyGGTbqBcqGGPbqAcqGGUbGBcqGGSaalcqqGbbqqaeqaaOGaeiikaGIaee4uamLaeiykaKcabaGaemOCai3aaSbaaSqaaiGbc2gaTjabcggaHjabcIha4jabcYcaSiabbgeabbqabaGccqGGOaakcqqGtbWucqGGPaqkcqGHsislcqWGYbGCdaWgaaWcbaGagiyBa0MaeiyAaKMaeiOBa4MaeiilaWIaeeyqaeeabeaakiabcIcaOiabbofatjabcMcaPaaaaaa@5FCA@

with

rmax⁡,A(S)=max⁡0≤A1≤A1max⁡0≤A2≤A2max⁡r(S,A1,A2)
 MathType@MTEF@5@5@+=feaafiart1ev1aaatCvAUfKttLearuWrP9MDH5MBPbIqV92AaeXatLxBI9gBaebbnrfifHhDYfgasaacH8akY=wiFfYdH8Gipec8Eeeu0xXdbba9frFj0=OqFfea0dXdd9vqai=hGuQ8kuc9pgc9s8qqaq=dirpe0xb9q8qiLsFr0=vr0=vr0dc8meaabaqaciaacaGaaeqabaqabeGadaaakeaacqWGYbGCdaWgaaWcbaGagiyBa0MaeiyyaeMaeiiEaGNaeiilaWIaeeyqaeeabeaakiabcIcaOiabbofatjabcMcaPiabg2da9maaxababaGagiyBa0MaeiyyaeMaeiiEaGhaleaafaqaaeGabaaabaGaeGimaaJaeyizImQaeeyqae0aaSbaaWqaaiabigdaXaqabaWccqGHKjYOcqqGbbqqdaWgaaadbaGaeGymaeZaaSbaaeaacyGGTbqBcqGGHbqycqGG4baEaeqaaaqabaaaleaacqaIWaamcqGHKjYOcqqGbbqqdaWgaaadbaGaeGOmaidabeaaliabgsMiJkabbgeabnaaBaaameaacqaIYaGmdaWgaaqaaiGbc2gaTjabcggaHjabcIha4bqabaaabeaaaaaaleqaaOGaemOCaiNaeiikaGIaee4uamLaeiilaWIaeeyqae0aaSbaaSqaaiabigdaXaqabaGccqGGSaalcqqGbbqqdaWgaaWcbaGaeGOmaidabeaakiabcMcaPaaa@612A@

rmin⁡,A(S)=min⁡0≤A1≤A1max⁡0≤A2≤A2max⁡r(S,A1,A2)
 MathType@MTEF@5@5@+=feaafiart1ev1aaatCvAUfKttLearuWrP9MDH5MBPbIqV92AaeXatLxBI9gBaebbnrfifHhDYfgasaacH8akY=wiFfYdH8Gipec8Eeeu0xXdbba9frFj0=OqFfea0dXdd9vqai=hGuQ8kuc9pgc9s8qqaq=dirpe0xb9q8qiLsFr0=vr0=vr0dc8meaabaqaciaacaGaaeqabaqabeGadaaakeaacqWGYbGCdaWgaaWcbaGagiyBa0MaeiyAaKMaeiOBa4MaeiilaWIaeeyqaeeabeaakiabcIcaOiabbofatjabcMcaPiabg2da9maaxababaGagiyBa0MaeiyAaKMaeiOBa4galeaafaqaaeGabaaabaGaeGimaaJaeyizImQaeeyqae0aaSbaaWqaaiabigdaXaqabaWccqGHKjYOcqqGbbqqdaWgaaadbaGaeGymaeZaaSbaaeaacyGGTbqBcqGGHbqycqGG4baEaeqaaaqabaaaleaacqaIWaamcqGHKjYOcqqGbbqqdaWgaaadbaGaeGOmaidabeaaliabgsMiJkabbgeabnaaBaaameaacqaIYaGmdaWgaaqaaiGbc2gaTjabcggaHjabcIha4bqabaaabeaaaaaaleqaaOGaemOCaiNaeiikaGIaee4uamLaeiilaWIaeeyqae0aaSbaaSqaaiabigdaXaqabaGccqGGSaalcqqGbbqqdaWgaaWcbaGaeGOmaidabeaakiabcMcaPaaa@6122@

The measure defined in Equation 14 gives an indication of the combined effect of both regulators and, hence, is denoted by 'resulting RS' *ν*_res _in the following.

Considerably more interesting than the combined effect *ν*_res _are the single influences of each activator, i.e. to what extent is the reaction activated by A_1 _and A_2_. Having the already-introduced approach in mind, such a quantification is possible by carrying out a limit calculation for only one effector while all other effectors are excluded (i.e. concentration is set to zero). The corresponding RS for the activator A_1 _is then defined as

νA1=r(S,A1,A2=0)−rmin⁡,A(S)rmax⁡,A1(S)−rmin⁡,A(S)
 MathType@MTEF@5@5@+=feaafiart1ev1aaatCvAUfKttLearuWrP9MDH5MBPbIqV92AaeXatLxBI9gBaebbnrfifHhDYfgasaacH8akY=wiFfYdH8Gipec8Eeeu0xXdbba9frFj0=OqFfea0dXdd9vqai=hGuQ8kuc9pgc9s8qqaq=dirpe0xb9q8qiLsFr0=vr0=vr0dc8meaabaqaciaacaGaaeqabaqabeGadaaakeaaiiGacqWF9oGBdaWgaaWcbaGaemyqae0aaSbaaWqaaiabigdaXaqabaaaleqaaOGaeyypa0ZaaSaaaeaacqWGYbGCcqGGOaakcqqGtbWucqGGSaalcqqGbbqqdaWgaaWcbaGaeGymaedabeaakiabcYcaSiabbgeabnaaBaaaleaacqaIYaGmaeqaaOGaeyypa0JaeGimaaJaeiykaKIaeyOeI0IaemOCai3aaSbaaSqaaiGbc2gaTjabcMgaPjabc6gaUjabcYcaSiabbgeabbqabaGccqGGOaakcqqGtbWucqGGPaqkaeaacqWGYbGCdaWgaaWcbaGagiyBa0MaeiyyaeMaeiiEaGNaeiilaWIaeeyqae0aaSbaaWqaaiabigdaXaqabaaaleqaaOGaeiikaGIaee4uamLaeiykaKIaeyOeI0IaemOCai3aaSbaaSqaaiGbc2gaTjabcMgaPjabc6gaUjabcYcaSiabbgeabbqabaGccqGGOaakcqqGtbWucqGGPaqkaaaaaa@60F7@

with

rmax⁡,A1(S)=max⁡0≤A1≤A1max⁡r(S,A2=0)
 MathType@MTEF@5@5@+=feaafiart1ev1aaatCvAUfKttLearuWrP9MDH5MBPbIqV92AaeXatLxBI9gBaebbnrfifHhDYfgasaacH8akY=wiFfYdH8Gipec8Eeeu0xXdbba9frFj0=OqFfea0dXdd9vqai=hGuQ8kuc9pgc9s8qqaq=dirpe0xb9q8qiLsFr0=vr0=vr0dc8meaabaqaciaacaGaaeqabaqabeGadaaakeaacqWGYbGCdaWgaaWcbaGagiyBa0MaeiyyaeMaeiiEaGNaeiilaWIaeeyqae0aaSbaaWqaaiabigdaXaqabaaaleqaaOGaeiikaGIaee4uamLaeiykaKIaeyypa0ZaaCbeaeaacyGGTbqBcqGGHbqycqGG4baEaSqaaiabicdaWiabgsMiJkabbgeabnaaBaaameaacqaIXaqmaeqaaSGaeyizImQaeeyqae0aaSbaaWqaaiabigdaXmaaBaaabaGagiyBa0MaeiyyaeMaeiiEaGhabeaaaeqaaaWcbeaakiabdkhaYjabcIcaOiabbofatjabcYcaSiabbgeabnaaBaaaleaacqaIYaGmaeqaaOGaeyypa0JaeGimaaJaeiykaKcaaa@5426@

By using Equation 17, the implicit assumption is made that each activator acts independently in the reaction. This is not true for the kinetic expression given in this example. Consequently, the single influences do not sum up to the resulting RS and, hence, an activating effect not equal to 100% can be obtained. A meaningful interpretation of such RS values is impossible and, therefore, a linear scaling of the single influences taking the *ν*_res _value into account is applied:

νres=ω(νA1+νA2)⇒ω=νresνA1+νA2
 MathType@MTEF@5@5@+=feaafiart1ev1aaatCvAUfKttLearuWrP9MDH5MBPbIqV92AaeXatLxBI9gBaebbnrfifHhDYfgasaacH8akY=wiFfYdH8Gipec8Eeeu0xXdbba9frFj0=OqFfea0dXdd9vqai=hGuQ8kuc9pgc9s8qqaq=dirpe0xb9q8qiLsFr0=vr0=vr0dc8meaabaqaciaacaGaaeqabaqabeGadaaakeaaiiGacqWF9oGBdaWgaaWcbaGaeeOCaiNaeeyzauMaee4Camhabeaakiabg2da9iab=L8a3jabcIcaOiab=17aUnaaBaaaleaacqqGbbqqdaWgaaadbaGaeGymaedabeaaaSqabaGccqGHRaWkcqWF9oGBdaWgaaWcbaGaeeyqae0aaSbaaWqaaiabikdaYaqabaaaleqaaOGaeiykaKIaeyO0H4Tae8xYdCNaeyypa0ZaaSaaaeaacqWF9oGBdaWgaaWcbaGaeeOCaiNaeeyzauMaee4CamhabeaaaOqaaiab=17aUnaaBaaaleaacqqGbbqqdaWgaaadbaGaeGymaedabeaaaSqabaGccqGHRaWkcqWF9oGBdaWgaaWcbaGaeeyqae0aaSbaaWqaaiabikdaYaqabaaaleqaaaaaaaa@54BD@

Using the determined scaling factor *ω *the single RS values are now defined as

ν˜Ai=ω⋅νAi,i=1,2
 MathType@MTEF@5@5@+=feaafiart1ev1aaatCvAUfKttLearuWrP9MDH5MBPbIqV92AaeXatLxBI9gBaebbnrfifHhDYfgasaacH8akY=wiFfYdH8Gipec8Eeeu0xXdbba9frFj0=OqFfea0dXdd9vqai=hGuQ8kuc9pgc9s8qqaq=dirpe0xb9q8qiLsFr0=vr0=vr0dc8meaabaqaciaacaGaaeqabaqabeGadaaakeaafaqabeqacaaabaacciGaf8xVd4MbaGaadaWgaaWcbaGaeeyqae0aaSbaaWqaaiabdMgaPbqabaaaleqaaOGaeyypa0Jae8xYdCNaeyyXICTae8xVd42aaSbaaSqaaiabbgeabnaaBaaameaacqWGPbqAaeqaaaWcbeaakiabcYcaSaqaaiabdMgaPjabg2da9iabigdaXiabcYcaSiabikdaYaaaaaa@40F9@

This simple form of a scaling can be applied in the case of reaction kinetics influenced by multiple inhibitors or activators.

#### Multiple effectors of oppositely directed influence

For the general case of an enzyme regulated by many inhibitors and activators, the already-introduced approach has to be extended once more. We show that a separate quantification of the influences of all participating effectors is possible by some simplifications, i.e. neglecting cross-interactions between effectors. Consider again the system shown in Figure 3 and the corresponding expression for the velocity rate in Equation 5.

The determination of *ν*_res _is analogous to Equation 14 based on the following definitions:

rmax⁡,e(S)=max⁡0≤A≤Amax⁡0≤I≤Imax⁡r(S,A,I)
 MathType@MTEF@5@5@+=feaafiart1ev1aaatCvAUfKttLearuWrP9MDH5MBPbIqV92AaeXatLxBI9gBaebbnrfifHhDYfgasaacH8akY=wiFfYdH8Gipec8Eeeu0xXdbba9frFj0=OqFfea0dXdd9vqai=hGuQ8kuc9pgc9s8qqaq=dirpe0xb9q8qiLsFr0=vr0=vr0dc8meaabaqaciaacaGaaeqabaqabeGadaaakeaacqWGYbGCdaWgaaWcbaGagiyBa0MaeiyyaeMaeiiEaGNaeiilaWccbeGae8xzaugabeaakiabcIcaOiabbofatjabcMcaPiabg2da9maaxababaGagiyBa0MaeiyyaeMaeiiEaGhaleaafaqaaeGabaaabaGaeGimaaJaeyizImQaeeyqaeKaeyizImQaeeyqae0aaSbaaWqaaiGbc2gaTjabcggaHjabcIha4bqabaaaleaacqaIWaamcqGHKjYOcqqGjbqscqGHKjYOcqqGjbqsdaWgaaadbaGagiyBa0MaeiyyaeMaeiiEaGhabeaaaaaaleqaaOGaemOCaiNaeiikaGIaee4uamLaeiilaWIaeeyqaeKaeiilaWIaeeysaKKaeiykaKcaaa@5AE6@

rmin⁡,e(S)=min⁡0≤A≤Amax⁡0≤I≤Imax⁡r(S,A,I)
 MathType@MTEF@5@5@+=feaafiart1ev1aaatCvAUfKttLearuWrP9MDH5MBPbIqV92AaeXatLxBI9gBaebbnrfifHhDYfgasaacH8akY=wiFfYdH8Gipec8Eeeu0xXdbba9frFj0=OqFfea0dXdd9vqai=hGuQ8kuc9pgc9s8qqaq=dirpe0xb9q8qiLsFr0=vr0=vr0dc8meaabaqaciaacaGaaeqabaqabeGadaaakeaacqWGYbGCdaWgaaWcbaGagiyBa0MaeiyAaKMaeiOBa4MaeiilaWccbeGae8xzaugabeaakiabcIcaOiabbofatjabcMcaPiabg2da9maaxababaGagiyBa0MaeiyAaKMaeiOBa4galeaafaqaaeGabaaabaGaeGimaaJaeyizImQaeeyqaeKaeyizImQaeeyqae0aaSbaaWqaaiGbc2gaTjabcggaHjabcIha4bqabaaaleaacqaIWaamcqGHKjYOcqqGjbqscqGHKjYOcqqGjbqsdaWgaaadbaGagiyBa0MaeiyyaeMaeiiEaGhabeaaaaaaleqaaOGaemOCaiNaeiikaGIaee4uamLaeiilaWIaeeyqaeKaeiilaWIaeeysaKKaeiykaKcaaa@5ADE@

**Figure 4:**
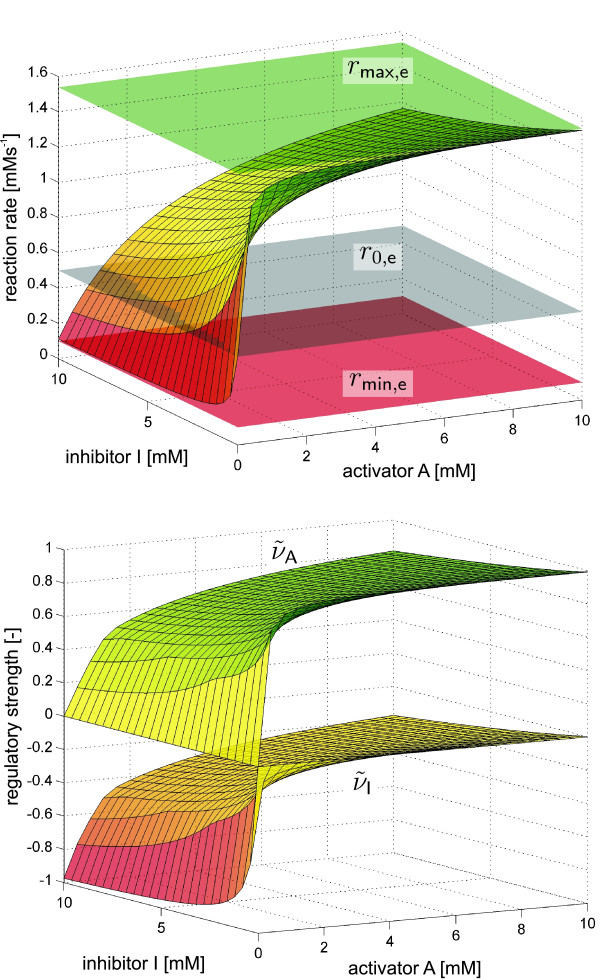
**Example kinetics of Michaelis-Menten type comprising one S, A and I pool**. The two effectors are competitive with respect to each other. Top: The combined regulatory effect (*ν*_res_) is determined by scaling of the present flux. Bottom: Using *ν*_res _the scaled single effctor influences of the activator (ν˜A
 MathType@MTEF@5@5@+=feaafiart1ev1aaatCvAUfKttLearuWrP9MDH5MBPbIqV92AaeXatLxBI9gBaebbnrfifHhDYfgasaacH8akY=wiFfYdH8Gipec8Eeeu0xXdbba9frFj0=OqFfea0dXdd9vqai=hGuQ8kuc9pgc9s8qqaq=dirpe0xb9q8qiLsFr0=vr0=vr0dc8meaabaqaciaacaGaaeqabaqabeGadaaakeaaiiGacuWF9oGBgaacamaaBaaaleaacqqGbbqqaeqaaaaa@2FAF@) and the inhibitor (ν˜I
 MathType@MTEF@5@5@+=feaafiart1ev1aaatCvAUfKttLearuWrP9MDH5MBPbIqV92AaeXatLxBI9gBaebbnrfifHhDYfgasaacH8akY=wiFfYdH8Gipec8Eeeu0xXdbba9frFj0=OqFfea0dXdd9vqai=hGuQ8kuc9pgc9s8qqaq=dirpe0xb9q8qiLsFr0=vr0=vr0dc8meaabaqaciaacaGaaeqabaqabeGadaaakeaaiiGacuWF9oGBgaacamaaBaaaleaacqqGjbqsaeqaaaaa@2FBF@) can be determined.

Owing to the opposite effect of the activator and inhibitor, the reaction rate runs between two scaling boundaries that indicate a maximal activation *r*_max, e_(S) or inhibition *r*_min, e_(S). Hence, for the calculation of *ν*_res _a flux value for the completely non-regulated state must be defined (cf. Figure 4):

r0,e(S)=max⁡0≤I≤Imax⁡min⁡0≤A≤Amax⁡r(S,A,I)
 MathType@MTEF@5@5@+=feaafiart1ev1aaatCvAUfKttLearuWrP9MDH5MBPbIqV92AaeXatLxBI9gBaebbnrfifHhDYfgasaacH8akY=wiFfYdH8Gipec8Eeeu0xXdbba9frFj0=OqFfea0dXdd9vqai=hGuQ8kuc9pgc9s8qqaq=dirpe0xb9q8qiLsFr0=vr0=vr0dc8meaabaqaciaacaGaaeqabaqabeGadaaakeaacqWGYbGCdaWgaaWcbaGaeGimaaJaeiilaWccbeGae8xzaugabeaakiabcIcaOiabbofatjabcMcaPiabg2da9maaxababaGagiyBa0MaeiyyaeMaeiiEaGhaleaacqaIWaamcqGHKjYOcqqGjbqscqGHKjYOcqqGjbqsdaWgaaadbaGagiyBa0MaeiyyaeMaeiiEaGhabeaaaSqabaGcdaWfqaqaaiGbc2gaTjabcMgaPjabc6gaUbWcbaGaeGimaaJaeyizImQaeeyqaeKaeyizImQaeeyqae0aaSbaaWqaaiGbc2gaTjabcggaHjabcIha4bqabaaaleqaaOGaemOCaiNaeiikaGIaee4uamLaeiilaWIaeeyqaeKaeiilaWIaeeysaKKaeiykaKcaaa@5C07@

To decide whether the resulting RS of each effector is activating or inhibiting, the present reaction rate *r*(S, A, I) is compared with the rate without any regulatory influence:

νres={r(S,A,I)−r0,e(S)rmax⁡,e(S)−r0,e(S),r(S,A,I)≥r0,e(S)r(S,A,I)−r0,e(S)r0,e(S)−rmin⁡,e(S),r(S,A,I)<r0,e(S)
 MathType@MTEF@5@5@+=feaafiart1ev1aaatCvAUfKttLearuWrP9MDH5MBPbIqV92AaeXatLxBI9gBaebbnrfifHhDYfgasaacH8akY=wiFfYdH8Gipec8Eeeu0xXdbba9frFj0=OqFfea0dXdd9vqai=hGuQ8kuc9pgc9s8qqaq=dirpe0xb9q8qiLsFr0=vr0=vr0dc8meaabaqaciaacaGaaeqabaqabeGadaaakeaaiiGacqWF9oGBdaWgaaWcbaGaeeOCaiNaeeyzauMaee4Camhabeaakiabg2da9maaceqabaqbaeqabiGaaaqaamaalaaabaGaemOCaiNaeiikaGIaee4uamLaeiilaWIaeeyqaeKaeiilaWIaeeysaKKaeiykaKIaeyOeI0IaemOCai3aaSbaaSqaaiabicdaWiabcYcaSGqabiab+vgaLbqabaGccqGGOaakcqqGtbWucqGGPaqkaeaacqWGYbGCdaWgaaWcbaGagiyBa0MaeiyyaeMaeiiEaGNaeiilaWIae4xzaugabeaakiabcIcaOiabbofatjabcMcaPiabgkHiTiabdkhaYnaaBaaaleaacqaIWaamcqGGSaalcqGFLbqzaeqaaOGaeiikaGIaee4uamLaeiykaKcaaiabcYcaSaqaaiabdkhaYjabcIcaOiabbofatjabcYcaSiabbgeabjabcYcaSiabbMeajjabcMcaPiabgwMiZkabdkhaYnaaBaaaleaacqaIWaamcqGGSaalcqGFLbqzaeqaaOGaeiikaGIaee4uamLaeiykaKcabaWaaSaaaeaacqWGYbGCcqGGOaakcqqGtbWucqGGSaalcqqGbbqqcqGGSaalcqqGjbqscqGGPaqkcqGHsislcqWGYbGCdaWgaaWcbaGaeGimaaJaeiilaWIae4xzaugabeaakiabcIcaOiabbofatjabcMcaPaqaaiabdkhaYnaaBaaaleaacqaIWaamcqGGSaalcqGFLbqzaeqaaOGaeiikaGIaee4uamLaeiykaKIaeyOeI0IaemOCai3aaSbaaSqaaiGbc2gaTjabcMgaPjabc6gaUjabcYcaSiab+vgaLbqabaGccqGGOaakcqqGtbWucqGGPaqkaaGaeiilaWcabaGaemOCaiNaeiikaGIaee4uamLaeiilaWIaeeyqaeKaeiilaWIaeeysaKKaeiykaKIaeyipaWJaemOCai3aaSbaaSqaaiabicdaWiabcYcaSiab+vgaLbqabaGccqGGOaakcqqGtbWucqGGPaqkaaaacaGL7baaaaa@A177@

A value of *r*(S, A, I) greater than *r*_0, **e**_(S) indicates a resulting activation of the reaction and, hence, the upper and lower scaling boundaries are set to *r*_max, **e**_(S) and *r*_0, **e**_(S), respectively. The RS of each single effector is determined by choosing a corresponding effector as variable while setting all other effector concentrations to zero. According to this, the RS value of the inhibitor I in the example system is defined as

νI=r(S,I,A=0)−r0,e(S)r0,e(S)−rmin⁡,I(S)
 MathType@MTEF@5@5@+=feaafiart1ev1aaatCvAUfKttLearuWrP9MDH5MBPbIqV92AaeXatLxBI9gBaebbnrfifHhDYfgasaacH8akY=wiFfYdH8Gipec8Eeeu0xXdbba9frFj0=OqFfea0dXdd9vqai=hGuQ8kuc9pgc9s8qqaq=dirpe0xb9q8qiLsFr0=vr0=vr0dc8meaabaqaciaacaGaaeqabaqabeGadaaakeaaiiGacqWF9oGBdaWgaaWcbaGaeeysaKeabeaakiabg2da9maalaaabaGaemOCaiNaeiikaGIaee4uamLaeiilaWIaeeysaKKaeiilaWIaeeyqaeKaeyypa0JaeGimaaJaeiykaKIaeyOeI0IaemOCai3aaSbaaSqaaiabicdaWiabcYcaSGqabiab+vgaLbqabaGccqGGOaakcqqGtbWucqGGPaqkaeaacqWGYbGCdaWgaaWcbaGaeGimaaJaeiilaWIae4xzaugabeaakiabcIcaOiabbofatjabcMcaPiabgkHiTiabdkhaYnaaBaaaleaacyGGTbqBcqGGPbqAcqGGUbGBcqGGSaalcqqGjbqsaeqaaOGaeiikaGIaee4uamLaeiykaKcaaaaa@56AF@

with

rmin⁡,I(S)=min⁡0≤I≤Imax⁡r(S,A=0)
 MathType@MTEF@5@5@+=feaafiart1ev1aaatCvAUfKttLearuWrP9MDH5MBPbIqV92AaeXatLxBI9gBaebbnrfifHhDYfgasaacH8akY=wiFfYdH8Gipec8Eeeu0xXdbba9frFj0=OqFfea0dXdd9vqai=hGuQ8kuc9pgc9s8qqaq=dirpe0xb9q8qiLsFr0=vr0=vr0dc8meaabaqaciaacaGaaeqabaqabeGadaaakeaacqWGYbGCdaWgaaWcbaGagiyBa0MaeiyAaKMaeiOBa4MaeiilaWIaeeysaKeabeaakiabcIcaOiabbofatjabcMcaPiabg2da9maaxababaGagiyBa0MaeiyAaKMaeiOBa4galeaacqaIWaamcqGHKjYOcqqGjbqscqGHKjYOcqqGjbqsdaWgaaadbaGagiyBa0MaeiyyaeMaeiiEaGhabeaaaSqabaGccqWGYbGCcqGGOaakcqqGtbWucqGGSaalcqqGbbqqcqGH9aqpcqaIWaamcqGGPaqkaaa@4FC5@

As already mentioned these definitions do not take the cross-interactions between the influences of different effectors into account. To approximately quantify these correlations a further scaling is applied, also considering the different effect of the activator and inhibitor:

νres=ω⋅νA+(1−ω)⋅νI⇒ω=νres−νIνA−νI
 MathType@MTEF@5@5@+=feaafiart1ev1aaatCvAUfKttLearuWrP9MDH5MBPbIqV92AaeXatLxBI9gBaebbnrfifHhDYfgasaacH8akY=wiFfYdH8Gipec8Eeeu0xXdbba9frFj0=OqFfea0dXdd9vqai=hGuQ8kuc9pgc9s8qqaq=dirpe0xb9q8qiLsFr0=vr0=vr0dc8meaabaqaciaacaGaaeqabaqabeGadaaakeaaiiGacqWF9oGBdaWgaaWcbaGaeeOCaiNaeeyzauMaee4Camhabeaakiabg2da9iab=L8a3jabgwSixlab=17aUnaaBaaaleaacqqGbbqqaeqaaOGaey4kaSIaeiikaGIaeGymaeJaey4kaSIae8xYdCNaeiykaKIaeyyXICTae8xVd42aaSbaaSqaaiabbMeajbqabaGccqGHshI3cqWFjpWDcqGH9aqpdaWcaaqaaiab=17aUnaaBaaaleaacqqGYbGCcqqGLbqzcqqGZbWCaeqaaOGaeyOeI0Iae8xVd42aaSbaaSqaaiabbMeajbqabaaakeaacqWF9oGBdaWgaaWcbaGaeeyqaeeabeaakiabgkHiTiab=17aUnaaBaaaleaacqqGjbqsaeqaaaaaaaa@5C61@

Finally, using the determined scaling factor *ω *the single influences are defined as

ν˜A=ω⋅νA,ν˜I=(1−ω)νI
 MathType@MTEF@5@5@+=feaafiart1ev1aaatCvAUfKttLearuWrP9MDH5MBPbIqV92AaeXatLxBI9gBaebbnrfifHhDYfgasaacH8akY=wiFfYdH8Gipec8Eeeu0xXdbba9frFj0=OqFfea0dXdd9vqai=hGuQ8kuc9pgc9s8qqaq=dirpe0xb9q8qiLsFr0=vr0=vr0dc8meaabaqaciaacaGaaeqabaqabeGadaaakeaafaqabeqacaaabaacciGaf8xVd4MbaGaadaWgaaWcbaGaeeyqaeeabeaakiabg2da9iab=L8a3jabgwSixlab=17aUnaaBaaaleaacqqGbbqqaeqaaOGaeiilaWcabaGaf8xVd4MbaGaadaWgaaWcbaGaeeysaKeabeaakiabg2da9iabcIcaOiabigdaXiabgkHiTiab=L8a3jabcMcaPiab=17aUnaaBaaaleaacqqGjbqsaeqaaaaaaaa@4516@

The resulting percentages ν˜A
 MathType@MTEF@5@5@+=feaafiart1ev1aaatCvAUfKttLearuWrP9MDH5MBPbIqV92AaeXatLxBI9gBaebbnrfifHhDYfgasaacH8akY=wiFfYdH8Gipec8Eeeu0xXdbba9frFj0=OqFfea0dXdd9vqai=hGuQ8kuc9pgc9s8qqaq=dirpe0xb9q8qiLsFr0=vr0=vr0dc8meaabaqaciaacaGaaeqabaqabeGadaaakeaaiiGacuWF9oGBgaacamaaBaaaleaacqqGbbqqaeqaaaaa@2FAF@ and ν˜I
 MathType@MTEF@5@5@+=feaafiart1ev1aaatCvAUfKttLearuWrP9MDH5MBPbIqV92AaeXatLxBI9gBaebbnrfifHhDYfgasaacH8akY=wiFfYdH8Gipec8Eeeu0xXdbba9frFj0=OqFfea0dXdd9vqai=hGuQ8kuc9pgc9s8qqaq=dirpe0xb9q8qiLsFr0=vr0=vr0dc8meaabaqaciaacaGaaeqabaqabeGadaaakeaaiiGacuWF9oGBgaacamaaBaaaleaacqqGjbqsaeqaaaaa@2FBF@ now indicate the proportion in which the different effectors contribute to the total regulation of the reaction step (cf. Figure 4).

At this point, it becomes clear that the calculated values for the single RS of each effector are not 'precise' values. However, for a graphical visualization, a quantity given in an approximate magnitude is adequate, unless the sign of the RS value corresponds to the underlying effect of the regulator (i.e. + activator or - inhibitor).

## Results and Discussion

### General definition of RS

In the following a general definition for the RS is exemplified by using a frequently used kinetic model often applied in dynamic metabolic network models [13,14]. The enzymatic conversion of PEP to Pyr as a reaction step within the glycolysis is catalyzed by the Pk enzyme. This reaction is allosterically activated by FBP and AMP as well as inhibited by ATP:

rPk=[rmax⁡PEP(PEPKPEP+1)(n−1)ADP]×[KPEP(L((1+ATPKATP)(FBPKFBP+AMPKAMP+1)−1)n+(PEPKPEP+1)n)(ADP+KADP)]−1
 MathType@MTEF@5@5@+=feaafiart1ev1aaatCvAUfKttLearuWrP9MDH5MBPbIqV92AaeXatLxBI9gBaebbnrfifHhDYfgasaacH8akY=wiFfYdH8Gipec8Eeeu0xXdbba9frFj0=OqFfea0dXdd9vqai=hGuQ8kuc9pgc9s8qqaq=dirpe0xb9q8qiLsFr0=vr0=vr0dc8meaabaqaciaacaGaaeqabaqabeGadaaakeaafaqadeGabaaabaGaemOCai3aaSbaaSqaaiabbcfaqjabbUgaRbqabaGccqGH9aqpdaWadaqaaiabdkhaYnaaBaaaleaacyGGTbqBcqGGHbqycqGG4baEaeqaaOGaeeiuaaLaeeyrauKaeeiuaa1aaeWaaeaadaWcaaqaaiabbcfaqjabbweafjabbcfaqbqaaiabbUealnaaBaaaleaacqqGqbaucqqGfbqrcqqGqbauaeqaaaaakiabgUcaRiabigdaXaGaayjkaiaawMcaamaaCaaaleqabaGaeiikaGIaemOBa4MaeyOeI0IaeGymaeJaeiykaKcaaOGaeeyqaeKaeeiraqKaeeiuaafacaGLBbGaayzxaaaabaGaey41aq7aamWaaeaacqqGlbWsdaWgaaWcbaGaeeiuaaLaeeyrauKaeeiuaafabeaakmaabmaabaGaemitaW0aaeWaaeaadaqadaqaaiabigdaXiabgUcaRmaalaaabaGaeeyqaeKaeeivaqLaeeiuaafabaGaee4saS0aaSbaaSqaaiabbgeabjabbsfaujabbcfaqbqabaaaaaGccaGLOaGaayzkaaWaaeWaaeaadaWcaaqaaiabbAeagjabbkeacjabbcfaqbqaaiabbUealnaaBaaaleaacqqGgbGrcqqGcbGqcqqGqbauaeqaaaaakiabgUcaRmaalaaabaGaeeyqaeKaeeyta0KaeeiuaafabaGaee4saS0aaSbaaSqaaiabbgeabjabb2eanjabbcfaqbqabaaaaOGaey4kaSIaeGymaedacaGLOaGaayzkaaWaaWbaaSqabeaacqGHsislcqaIXaqmaaaakiaawIcacaGLPaaadaahaaWcbeqaaiabd6gaUbaakiabgUcaRmaabmaabaWaaSaaaeaacqqGqbaucqqGfbqrcqqGqbauaeaacqqGlbWsdaWgaaWcbaGaeeiuaaLaeeyrauKaeeiuaafabeaaaaGccqGHRaWkcqaIXaqmaiaawIcacaGLPaaadaahaaWcbeqaaiabd6gaUbaaaOGaayjkaiaawMcaamaabmaabaGaeeyqaeKaeeiraqKaeeiuaaLaey4kaSIaee4saS0aaSbaaSqaaiabbgeabjabbseaejabbcfaqbqabaaakiaawIcacaGLPaaaaiaawUfacaGLDbaadaahaaWcbeqaaiabgkHiTiabigdaXaaaaaaaaa@9B71@

The vectors **s **= [PEP, ADP]^T ^and **e **= [ATP, FBP, AMP]^T ^comprise the substrate and effector concentrations included in the Pk reaction. The necessary steps for determining the RS values of all effectors are as follows.

1. Separation of all effectors into activators **a **= [FBP, AMP]^T ^and inhibitors **i **= [ATP].

2. Definition of maximal effector concentrations **a**_max _= [FBP_max_, AMP_max_]^T ^and **i**_max _= [ATP_max_].

3. Determination of the resulting RS:

νres={r(s,e)−r0,e(s)rmax⁡,e(s)−r0,e(s),r(s,e)≥r0,e(s)r(s,e)−r0,e(s)r0,e(s)−rmin⁡,e(s),r(s,e)<r0,e(s)
 MathType@MTEF@5@5@+=feaafiart1ev1aaatCvAUfKttLearuWrP9MDH5MBPbIqV92AaeXatLxBI9gBaebbnrfifHhDYfgasaacH8akY=wiFfYdH8Gipec8Eeeu0xXdbba9frFj0=OqFfea0dXdd9vqai=hGuQ8kuc9pgc9s8qqaq=dirpe0xb9q8qiLsFr0=vr0=vr0dc8meaabaqaciaacaGaaeqabaqabeGadaaakeaaiiGacqWF9oGBdaWgaaWcbaGaeeOCaiNaeeyzauMaee4Camhabeaakiabg2da9maaceqabaqbaeaabiGaaaqaamaalaaabaGaemOCaiNaeiikaGccbeGae43CamNaeiilaWIae4xzauMaeiykaKIaeyOeI0IaemOCai3aaSbaaSqaaiabicdaWiabcYcaSiab+vgaLbqabaGccqGGOaakcqGFZbWCcqGGPaqkaeaacqWGYbGCdaWgaaWcbaGagiyBa0MaeiyyaeMaeiiEaGNaeiilaWIae4xzaugabeaakiabcIcaOiab+nhaZjabcMcaPiabgkHiTiabdkhaYnaaBaaaleaacqaIWaamcqGGSaalcqGFLbqzaeqaaOGaeiikaGIae43CamNaeiykaKcaaiabcYcaSaqaaiabdkhaYjabcIcaOiab+nhaZjabcYcaSiab+vgaLjabcMcaPiabgwMiZkabdkhaYnaaBaaaleaacqaIWaamcqGGSaalcqGFLbqzaeqaaOGaeiikaGIae43CamNaeiykaKcabaWaaSaaaeaacqWGYbGCcqGGOaakcqGFZbWCcqGGSaalcqGFLbqzcqGGPaqkcqGHsislcqWGYbGCdaWgaaWcbaGaeGimaaJaeiilaWIae4xzaugabeaakiabcIcaOiab+nhaZjabcMcaPaqaaiabdkhaYnaaBaaaleaacqaIWaamcqGGSaalcqGFLbqzaeqaaOGaeiikaGIae43CamNaeiykaKIaeyOeI0IaemOCai3aaSbaaSqaaiGbc2gaTjabcMgaPjabc6gaUjabcYcaSiab+vgaLbqabaGccqGGOaakcqGFZbWCcqGGPaqkaaGaeiilaWcabaGaemOCaiNaeiikaGIae43CamNaeiilaWIae4xzauMaeiykaKIaeyipaWJaemOCai3aaSbaaSqaaiabicdaWiabcYcaSiab+vgaLbqabaGccqGGOaakcqGFZbWCcqGGPaqkaaaacaGL7baaaaa@9D82@

with

r0,e(s)=max⁡0≤i≤imax⁡min⁡0≤a≤amax⁡r(s,e)
 MathType@MTEF@5@5@+=feaafiart1ev1aaatCvAUfKttLearuWrP9MDH5MBPbIqV92AaeXatLxBI9gBaebbnrfifHhDYfgasaacH8akY=wiFfYdH8Gipec8Eeeu0xXdbba9frFj0=OqFfea0dXdd9vqai=hGuQ8kuc9pgc9s8qqaq=dirpe0xb9q8qiLsFr0=vr0=vr0dc8meaabaqaciaacaGaaeqabaqabeGadaaakeaacqWGYbGCdaWgaaWcbaGaeGimaaJaeiilaWccbeGae8xzaugabeaakiabcIcaOiab=nhaZjabcMcaPiabg2da9maaxababaGagiyBa0MaeiyyaeMaeiiEaGhaleaacqaIWaamcqGHKjYOcqWFPbqAcqGHKjYOcqWFPbqAdaWgaaadbaGagiyBa0MaeiyyaeMaeiiEaGhabeaaaSqabaGcdaWfqaqaaiGbc2gaTjabcMgaPjabc6gaUbWcbaGaeGimaaJaeyizImQae8xyaeMaeyizImQae8xyae2aaSbaaWqaaiGbc2gaTjabcggaHjabcIha4bqabaaaleqaaOGaemOCaiNaeiikaGIae83CamNaeiilaWIae8xzauMaeiykaKcaaa@5BC8@

rmax⁡,e(s)=max⁡0≤e≤emax⁡r(s,e)
 MathType@MTEF@5@5@+=feaafiart1ev1aaatCvAUfKttLearuWrP9MDH5MBPbIqV92AaeXatLxBI9gBaebbnrfifHhDYfgasaacH8akY=wiFfYdH8Gipec8Eeeu0xXdbba9frFj0=OqFfea0dXdd9vqai=hGuQ8kuc9pgc9s8qqaq=dirpe0xb9q8qiLsFr0=vr0=vr0dc8meaabaqaciaacaGaaeqabaqabeGadaaakeaacqWGYbGCdaWgaaWcbaGagiyBa0MaeiyyaeMaeiiEaGNaeiilaWccbeGae8xzaugabeaakiabcIcaOiab=nhaZjabcMcaPiabg2da9maaxababaGagiyBa0MaeiyyaeMaeiiEaGhaleaacqaIWaamcqGHKjYOcqWFLbqzcqGHKjYOcqWFLbqzdaWgaaadbaGagiyBa0MaeiyyaeMaeiiEaGhabeaaaSqabaGccqWGYbGCcqGGOaakcqWFZbWCcqGGSaalcqWFLbqzcqGGPaqkaaa@4F47@

rmin⁡,e(s)=min⁡0≤e≤emax⁡r(s,e)
 MathType@MTEF@5@5@+=feaafiart1ev1aaatCvAUfKttLearuWrP9MDH5MBPbIqV92AaeXatLxBI9gBaebbnrfifHhDYfgasaacH8akY=wiFfYdH8Gipec8Eeeu0xXdbba9frFj0=OqFfea0dXdd9vqai=hGuQ8kuc9pgc9s8qqaq=dirpe0xb9q8qiLsFr0=vr0=vr0dc8meaabaqaciaacaGaaeqabaqabeGadaaakeaacqWGYbGCdaWgaaWcbaGagiyBa0MaeiyAaKMaeiOBa4MaeiilaWccbeGae8xzaugabeaakiabcIcaOiab=nhaZjabcMcaPiabg2da9maaxababaGagiyBa0MaeiyAaKMaeiOBa4galeaacqaIWaamcqGHKjYOcqWFLbqzcqGHKjYOcqWFLbqzdaWgaaadbaGagiyBa0MaeiyyaeMaeiiEaGhabeaaaSqabaGccqWGYbGCcqGGOaakcqWFZbWCcqGGSaalcqWFLbqzcqGGPaqkaaa@4F3F@

4. Determination of single effector influences:

νek=r(s,0︸e1,...,0︸ek−1,ek,0︸ek+1,...,0︸en)−r0,e(s)|rek(s)−r0,e(s)|
 MathType@MTEF@5@5@+=feaafiart1ev1aaatCvAUfKttLearuWrP9MDH5MBPbIqV92AaeXatLxBI9gBaebbnrfifHhDYfgasaacH8akY=wiFfYdH8Gipec8Eeeu0xXdbba9frFj0=OqFfea0dXdd9vqai=hGuQ8kuc9pgc9s8qqaq=dirpe0xb9q8qiLsFr0=vr0=vr0dc8meaabaqaciaacaGaaeqabaqabeGadaaakeaaiiGacqWF9oGBdaWgaaWcbaGaemyzau2aaSbaaWqaaiabdUgaRbqabaaaleqaaOGaeyypa0ZaaSaaaeaacqWGYbGCcqGGOaakieqacqGFZbWCcqGGSaaldaagaaqaaiabicdaWaWcbaGaemyzau2aaSbaaWqaaiabigdaXaqabaaakiaawIJ=aiabcYcaSiabc6caUiabc6caUiabc6caUiabcYcaSmaayaaabaGaeGimaadaleaacqWGLbqzdaWgaaadbaGaem4AaSMaeyOeI0IaeGymaedabeaaaOGaayjo+dGaeiilaWIaemyzau2aaSbaaSqaaiabdUgaRbqabaGccqGGSaaldaagaaqaaiabicdaWaWcbaGaemyzau2aaSbaaWqaaiabdUgaRjabgUcaRiabigdaXaqabaaakiaawIJ=aiabcYcaSiabc6caUiabc6caUiabc6caUiabcYcaSmaayaaabaGaeGimaadaleaacqWGLbqzdaWgaaadbaGaemOBa4gabeaaaOGaayjo+dGaeiykaKIaeyOeI0IaemOCai3aaSbaaSqaaiabicdaWiabcYcaSiab+vgaLbqabaGccqGGOaakcqGFZbWCcqGGPaqkaeaadaabdaqaaiabdkhaYnaaBaaaleaacqWGLbqzdaWgaaadbaGaem4AaSgabeaaaSqabaGccqGGOaakcqGFZbWCcqGGPaqkcqGHsislcqWGYbGCdaWgaaWcbaGaeGimaaJaeiilaWIae4xzaugabeaakiabcIcaOiab+nhaZjabcMcaPaGaay5bSlaawIa7aaaaaaa@7C69@

with

rek(s)={max⁡0≤ek≤ekmax⁡r(s,0,...,0,ek,0,...,0),ek∈amin⁡0≤ek≤ekmax⁡r(s,0,...,0,ek,0,...,0),ek∈i
 MathType@MTEF@5@5@+=feaafiart1ev1aaatCvAUfKttLearuWrP9MDH5MBPbIqV92AaeXatLxBI9gBaebbnrfifHhDYfgasaacH8akY=wiFfYdH8Gipec8Eeeu0xXdbba9frFj0=OqFfea0dXdd9vqai=hGuQ8kuc9pgc9s8qqaq=dirpe0xb9q8qiLsFr0=vr0=vr0dc8meaabaqaciaacaGaaeqabaqabeGadaaakeaacqWGYbGCdaWgaaWcbaGaemyzau2aaSbaaWqaaiabdUgaRbqabaaaleqaaOGaeiikaGccbeGae83CamNaeiykaKIaeyypa0ZaaiqabeaafaqaaeGacaaabaGagiyBa0MaeiyyaeMaeiiEaG3aaSbaaSqaaiabicdaWiabgsMiJkabdwgaLnaaBaaameaacqWGRbWAaeqaaSGaeyizImQaemyzau2aaSbaaWqaaiabdUgaRnaaBaaabaGagiyBa0MaeiyyaeMaeiiEaGhabeaaaeqaaaWcbeaakiabdkhaYjabcIcaOiab=nhaZjabcYcaSiabicdaWiabcYcaSiabc6caUiabc6caUiabc6caUiabcYcaSiabicdaWiabcYcaSiabdwgaLnaaBaaaleaacqWGRbWAaeqaaOGaeiilaWIaeGimaaJaeiilaWIaeiOla4IaeiOla4IaeiOla4IaeiilaWIaeGimaaJaeiykaKIaeiilaWcabaGaemyzau2aaSbaaSqaaiabdUgaRbqabaGccqGHiiIZcqWFHbqyaeaacyGGTbqBcqGGPbqAcqGGUbGBdaWgaaWcbaGaeGimaaJaeyizImQaemyzau2aaSbaaWqaaiabdUgaRbqabaWccqGHKjYOcqWGLbqzdaWgaaadbaGaem4AaS2aaSbaaeaacyGGTbqBcqGGHbqycqGG4baEaeqaaaqabaaaleqaaOGaemOCaiNaeiikaGIae83CamNaeiilaWIaeGimaaJaeiilaWIaeiOla4IaeiOla4IaeiOla4IaeiilaWIaeGimaaJaeiilaWIaemyzau2aaSbaaSqaaiabdUgaRbqabaGccqGGSaalcqaIWaamcqGGSaalcqGGUaGlcqGGUaGlcqGGUaGlcqGGSaalcqaIWaamcqGGPaqkcqGGSaalaeaacqWGLbqzdaWgaaWcbaGaem4AaSgabeaakiabgIGiolab=LgaPbaaaiaawUhaaaaa@96A4@

5. Determination of scaled single effector influences:

ν˜ek={ω⋅νek,ek∈a(1−ω)νek,ek∈i
 MathType@MTEF@5@5@+=feaafiart1ev1aaatCvAUfKttLearuWrP9MDH5MBPbIqV92AaeXatLxBI9gBaebbnrfifHhDYfgasaacH8akY=wiFfYdH8Gipec8Eeeu0xXdbba9frFj0=OqFfea0dXdd9vqai=hGuQ8kuc9pgc9s8qqaq=dirpe0xb9q8qiLsFr0=vr0=vr0dc8meaabaqaciaacaGaaeqabaqabeGadaaakeaaiiGacuWF9oGBgaacamaaBaaaleaacqWGLbqzdaWgaaadbaGaem4AaSgabeaaaSqabaGccqGH9aqpdaGabeqaauaabaqaciaaaeaacqWFjpWDcqGHflY1cqWF9oGBdaWgaaWcbaGaemyzau2aaSbaaWqaaiabdUgaRbqabaaaleqaaOGaeiilaWcabaGaemyzau2aaSbaaSqaaiabdUgaRbqabaGccqGHiiIZieqacqGFHbqyaeaacqGGOaakcqaIXaqmcqGHsislcqWFjpWDcqGGPaqkcqWF9oGBdaWgaaWcbaGaemyzau2aaSbaaWqaaiabdUgaRbqabaaaleqaaOGaeiilaWcabaGaemyzau2aaSbaaSqaaiabdUgaRbqabaGccqGHiiIZcqGFPbqAaaaacaGL7baaaaa@5417@

with

ω=νres−∑νi∑νa−∑νi
 MathType@MTEF@5@5@+=feaafiart1ev1aaatCvAUfKttLearuWrP9MDH5MBPbIqV92AaeXatLxBI9gBaebbnrfifHhDYfgasaacH8akY=wiFfYdH8Gipec8Eeeu0xXdbba9frFj0=OqFfea0dXdd9vqai=hGuQ8kuc9pgc9s8qqaq=dirpe0xb9q8qiLsFr0=vr0=vr0dc8meaabaqaciaacaGaaeqabaqabeGadaaakeaaiiGacqWFjpWDcqGH9aqpdaWcaaqaaiab=17aUnaaBaaaleaacqqGYbGCcqqGLbqzcqqGZbWCaeqaaOGaeyOeI0YaaabqaeaacqWF9oGBdaWgaaWcbaGaemyAaKgabeaaaeqabeqdcqGHris5aaGcbaWaaabqaeaacqWF9oGBdaWgaaWcbaGaemyyaegabeaakiabgkHiTmaaqaeabaGae8xVd42aaSbaaSqaaiabdMgaPbqabaaabeqab0GaeyyeIuoaaSqabeqaniabggHiLdaaaaaa@475A@

**Figure 5:**
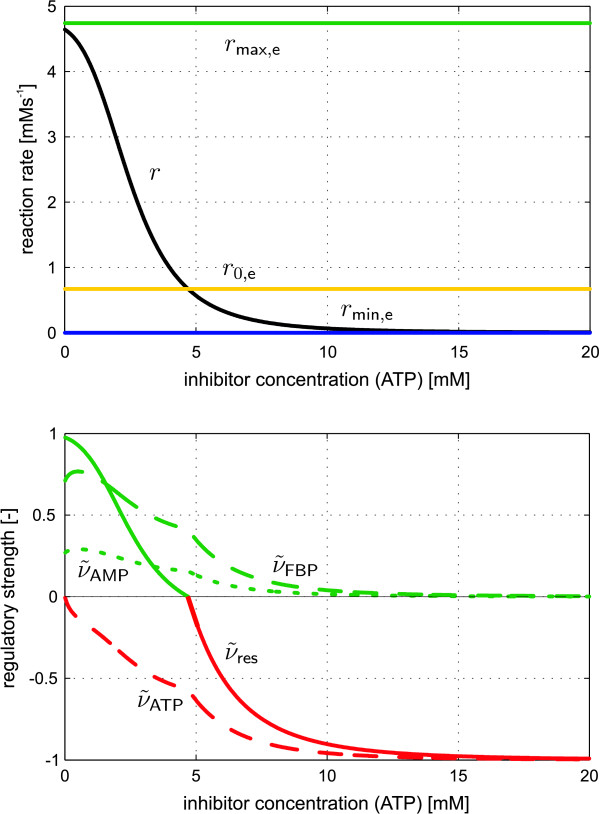
**RS determination of the Pk reaction**. The time courses of simulated reaction rates for the Pk reaction (top) are the basis for the determination of the resulting RS (*ν*_res_) as well as for single RS values ν˜ATP
 MathType@MTEF@5@5@+=feaafiart1ev1aaatCvAUfKttLearuWrP9MDH5MBPbIqV92AaeXatLxBI9gBaebbnrfifHhDYfgasaacH8akY=wiFfYdH8Gipec8Eeeu0xXdbba9frFj0=OqFfea0dXdd9vqai=hGuQ8kuc9pgc9s8qqaq=dirpe0xb9q8qiLsFr0=vr0=vr0dc8meaabaqaciaacaGaaeqabaqabeGadaaakeaaiiGacuWF9oGBgaacamaaBaaaleaacqqGbbqqcqqGubavcqqGqbauaeqaaaaa@3205@, ν˜FBP
 MathType@MTEF@5@5@+=feaafiart1ev1aaatCvAUfKttLearuWrP9MDH5MBPbIqV92AaeXatLxBI9gBaebbnrfifHhDYfgasaacH8akY=wiFfYdH8Gipec8Eeeu0xXdbba9frFj0=OqFfea0dXdd9vqai=hGuQ8kuc9pgc9s8qqaq=dirpe0xb9q8qiLsFr0=vr0=vr0dc8meaabaqaciaacaGaaeqabaqabeGadaaakeaaiiGacuWF9oGBgaacamaaBaaaleaacqqGgbGrcqqGcbGqcqqGqbauaeqaaaaa@31EB@, ν˜AMP
 MathType@MTEF@5@5@+=feaafiart1ev1aaatCvAUfKttLearuWrP9MDH5MBPbIqV92AaeXatLxBI9gBaebbnrfifHhDYfgasaacH8akY=wiFfYdH8Gipec8Eeeu0xXdbba9frFj0=OqFfea0dXdd9vqai=hGuQ8kuc9pgc9s8qqaq=dirpe0xb9q8qiLsFr0=vr0=vr0dc8meaabaqaciaacaGaaeqabaqabeGadaaakeaaiiGacuWF9oGBgaacamaaBaaaleaacqqGbbqqcqqGnbqtcqqGqbauaeqaaaaa@31F7@ of the inhibitor ATP and the two activators FBP and AMP, respectively (bottom). Kinetic parameters: ATP_max _= FBP_max _= AMP_max _= 10^8^; PEP = 0.8; ADP = FBP = 0.5; AMP = 0.1; *r*_max _= 10; *K*_PEP _= 0.31; *K*_ADP _= 0.26; *K*_ATP _= 1.5; *K*_FBP _= 0.19; *K*_AMP _= 0.2; *L *= 1000; *n *= 4.

Figure 5 shows the computed RS values of the kinetic expression for the Pk enzyme. Owing to the scaling applied (Equation 37) the single influences sum up to the resulting RS (*ν*_res_) of all effectors. Clearly, without any influence of the inhibitor ATP, the Pk reaction is activated. With increasing ATP concentration the RS of this effector also increases leading to a decrease of the Pk flux. At the same time the proportions (*ν*_FBP_, *ν*_AMP_) in which the two activators contribute to the total regulation are reduced. Above a value of ATP = 5 mM the regulation is solely determined by the inhibitor (i.e. *ν*_res _= *ν*_ATP _= 100%).

In order to allow for an automatic calculation of all RSs of a given metabolic network, the whole method is implemented in a Matlab GUI (version 7.2, supplied by The MathWorks Inc., Natick, MA) providing a direct interface to the MMT2 software package that is used for the simulation of dynamic network models [15]. Before starting the RS calculation, the effectors are classified and the upper bounds for all effectors are defined (items 1 and 2 of the general definition) based on the information from a preliminary simulation run. Afterwards the different range boundaries for arbitrary rate formulae, necessary for the RS determination, are sampled according to the time-dependent values of respective effector metabolites.

### Visualization tool

Along with this contribution, the network-based visualization tool MetVis has been extended for the visualization of RS data. MetVis was introduced in [8] as a tool for visualizing pool size and flux data under highly dynamic conditions. It represents pool sizes by level meters and fluxes by edge width. It also offers features for dynamic visualization and side-by-side network comparison.

A new feature of MetVis is the visualization of RS by edges connecting metabolite pools and reactions which are represented by nodes in a bipartite manner. Once the precise meaning of RS has been defined, the respective data can be generated by a simulation tool and used for visualization.

The results of simulations are usually delivered as a CSV structured file, containing information about the concentrations of metabolites, flows of reactions and the RS values of effectors. In the case of time-varying simulation data, the dynamic metabolic behavior contained in these data is expressed visually with an animation showing changing metabolite pool sizes and changing fluxes represented by differently filled boxes and varying arrow widths, respectively. Motivated by the fact that the metabolite concentrations and flux values can vary greatly, an adequate scaling of the input data is performed. This can be achieved in different ways depending on the scope of the study [16].

To visualize effectors using MetVis, additional edges representing the inhibition or activation effect connecting metabolites with reactions (enzymes) need to be inserted into the designed network. These connecting edges are visualized with a red circle for inhibition and a green circle for activation and are placed next to the affected reaction. The dynamic behavior of effector influences (i.e. the RS data) is displayed by changing the size of the corresponding circles indicating the level of the respective activation and inhibition.

### Visualization example

#### Dynamic network model

In the following the benefits and practical significance of the proposed visualization approach are illustrated with the help of a complex dynamic network model of the central carbon metabolism of *E. coli*, originally published in [14]. This network includes reactions for the glycolysis and the pentose phosphate pathway, which are linked via the sugar transport system (Pts).

The dynamic model is a system of ordinary differential equations consisting of 18 mass balance equations, 30 reaction rates and 7 analytical functions approximating the measured concentration values of cometabolites (AMP, NAD, etc.). All reactions steps are described by mechanistic enzyme kinetics resulting in a total number of 116 parameters. The special feature of this model is the high number of kinetic expressions describing regulatory interactions between metabolites and reactions (cf. Table 1), which makes it suitable as an example system. Simulation data of the network model were generated using the software package MMT2 [15].

**Table 1:** Regulatory information of the *E. coli *model.

Enzymatic reaction	Kinetic type	Activators	Inhibitors
Pts	-		G6P
Pgi	Reversible Michaelis-Menten		6PG
Pfk	Allosterical regulation	AMP, ADP	PEP
Pk	Allosterical regulation	FBP, AMP	ATP
PEPCx	Allosterical activation	FBP	
G1pat	Allosterical activation	FBP	
G6pdh	Irreversible Michaelis-Menten		NADPH
Pgdh	Irreversible Michaelis-Menten		NADPH, ATP

The table lists all enzyme kinetic expressions describing regulatory interactions.

#### Simulation results

**Figure 6:**
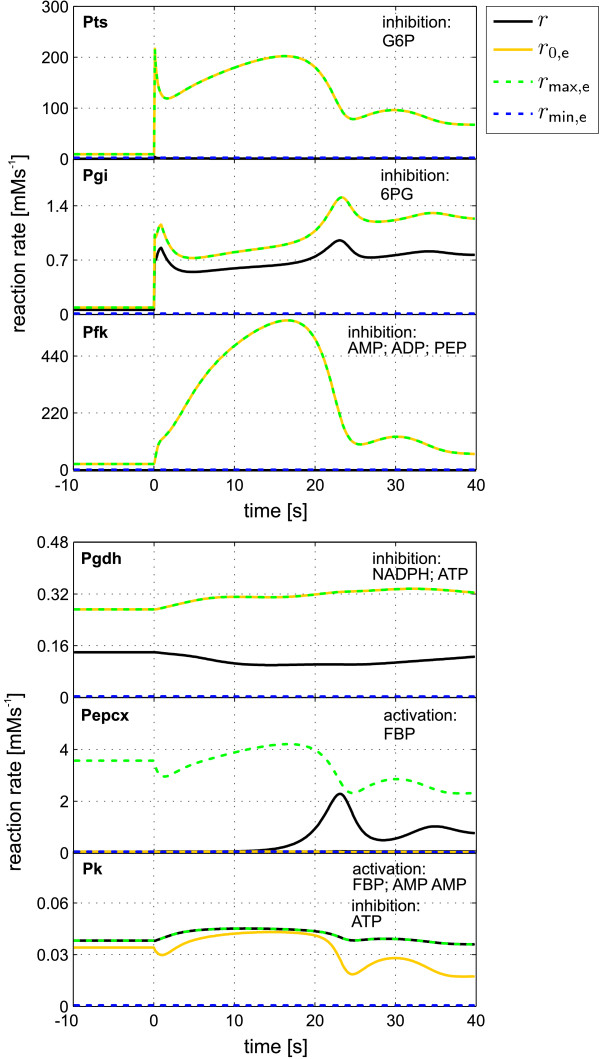
**RS determination of the *E. coli *model**. The plots show the time courses of simulated reaction rates for the determination of RS values. The scaling boundaries for the present flux *r *are denoted as *r*_0, e _for the flux in the completely non-regulated state, *r*_max, e _for the flux under maximal activation and *r*_min, e _for the flux under maximal inhibition.

**Figure 7:**
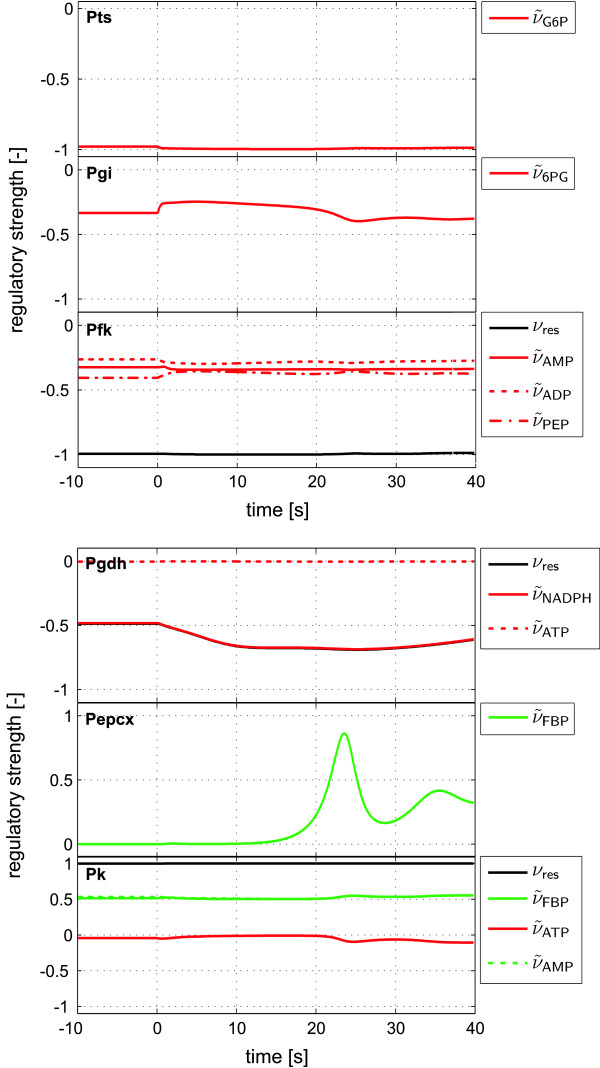
**Time courses of calculated RS values for the *E. coli *model**. For kinetic expressions comprising of only one effector the equality *ν*_res _= *ν*_e _holds.

By applying the presented approach to the example we simulated the influences of all effectors on the respective reaction steps. Figure 6 shows some of the reaction rates determined as in step 3 of the general algorithm. The resultant scaled single RS values are shown in Figure 7. By comparing the time-dependent RS data and the flux values, information on regulatory interactions within the metabolic network is immediately available.

• The RS values for the Pts reaction indicate a very strong inhibition by G6P in the stationary state as well as in the dynamic state after the glucose pulse. Comparing the two fluxes *r *and *r*_0, e _the enormous potential of an increase in the glucose transport in the case of an absent product inhibition can be clearly seen.

• The allosterically regulated enzyme Pfk is characterized by a nearly equal inhibition through all three effectors AMP, ADP and PEP. Interestingly, this is contradictory to the original definition of AMP and ADP having an activating influence on Pfk [17]. An explanation for this effect can be given by a closer look on the kinetic expression and the corresponding parameter values used in the model:

rPfk=rmax⁡ATP F6P(ATP+KATP(1+ADP/KADP,c))(F6P+KF6P(A/B))(1+C)A=1+ADPKADP,b+AMPKAMP,b+PEPKPEPB=1+ADPKADP,a+AMPKAMP,aC=L(1+F6P(B/KF6PA))n
 MathType@MTEF@5@5@+=feaafiart1ev1aaatCvAUfKttLearuWrP9MDH5MBPbIqV92AaeXatLxBI9gBaebbnrfifHhDYfgasaacH8akY=wiFfYdH8Gipec8Eeeu0xXdbba9frFj0=OqFfea0dXdd9vqai=hGuQ8kuc9pgc9s8qqaq=dirpe0xb9q8qiLsFr0=vr0=vr0dc8meaabaqaciaacaGaaeqabaqabeGadaaakeaafaqabeabbaaaaeaacqWGYbGCdaWgaaWcbaGaeeiuaaLaeeOzayMaee4AaSgabeaakiabg2da9maalaaabaGaemOCai3aaSbaaSqaaiGbc2gaTjabcggaHjabcIha4bqabaGccqqGbbqqcqqGubavcqqGqbaucqqGGaaicqqGgbGrcqqG2aGncqqGqbauaeaacqGGOaakcqqGbbqqcqqGubavcqqGqbaucqGHRaWkcqWGlbWsdaWgaaWcbaGaeeyqaeKaeeivaqLaeeiuaafabeaakiabcIcaOiabigdaXiabgUcaRiabbgeabjabbseaejabbcfaqjabc+caViabdUealnaaBaaaleaacqqGbbqqcqqGebarcqqGqbaucqGGSaalcqWGJbWyaeqaaOGaeiykaKIaeiykaKIaeiikaGIaeeOrayKaeeOnayJaeeiuaaLaey4kaSIaem4saS0aaSbaaSqaaiabbAeagjabbAda2iabbcfaqbqabaGccqGGOaakcqWGbbqqcqGGVaWlcqWGcbGqcqGGPaqkcqGGPaqkcqGGOaakcqaIXaqmcqGHRaWkcqWGdbWqcqGGPaqkaaaabaGaemyqaeKaeyypa0JaeGymaeJaey4kaSYaaSaaaeaacqqGbbqqcqqGebarcqqGqbauaeaacqWGlbWsdaWgaaWcbaGaeeyqaeKaeeiraqKaeeiuaaLaeiilaWIaemOyaigabeaaaaGccqGHRaWkdaWcaaqaaiabbgeabjabb2eanjabbcfaqbqaaiabdUealnaaBaaaleaacqqGbbqqcqqGnbqtcqqGqbaucqGGSaalcqWGIbGyaeqaaaaakiabgUcaRmaalaaabaGaeeiuaaLaeeyrauKaeeiuaafabaGaem4saS0aaSbaaSqaaiabbcfaqjabbweafjabbcfaqbqabaaaaaGcbaGaemOqaiKaeyypa0JaeGymaeJaey4kaSYaaSaaaeaacqqGbbqqcqqGebarcqqGqbauaeaacqWGlbWsdaWgaaWcbaGaeeyqaeKaeeiraqKaeeiuaaLaeiilaWIaemyyaegabeaaaaGccqGHRaWkdaWcaaqaaiabbgeabjabb2eanjabbcfaqbqaaiabdUealnaaBaaaleaacqqGbbqqcqqGnbqtcqqGqbaucqGGSaalcqWGHbqyaeqaaaaaaOqaaiabdoeadjabg2da9maalaaabaGaemitaWeabaGaeiikaGIaeGymaeJaey4kaSIaeeOrayKaeeOnayJaeeiuaaLaeiikaGIaemOqaiKaei4la8Iaem4saS0aaSbaaSqaaiabbAeagjabbAda2iabbcfaqbqabaGccqWGbbqqcqGGPaqkcqGGPaqkdaahaaWcbeqaaiabd6gaUbaaaaaaaaaa@BCD2@

Using the '*in vivo*' estimated parameter values *K*_ADP, *a *_= 128 mM, *K*_AMP, *a *_= 19.1 mM, *K*_ADP, *b *_= 3.89 mM, *K*_AMP, *b *_= 3.2 mM, it follows that *A *> *B *and, hence, an inhibitory influence of ADP and AMP is indeed present.

• The low activity of the Pk enzyme is solely determined by the generally small value of the maximal reaction rate (*r*_max _= 0.06 mM s^-1^) as the resulting effector influence (*ν*_res_) shows a strong activation. The same holds true for the G1pat reaction (cf. Figure 7), where FBP is a strong activator, but the enzyme activity is very low (*r*_max _= 0.008 mM s^-1^).

• The PEPCx reaction is described by a kinetic expression where the flux continuously increases with increasing concentration of the activator FBP. In this case the highest possible concentration for the effector is set slightly above the simulated maximum. Accordingly, the curves for *r *and *r*_max,e _nearly conjoined at this time point.

#### Network visualization

##### Visual interpretation of simulation data

**Figure 8:**
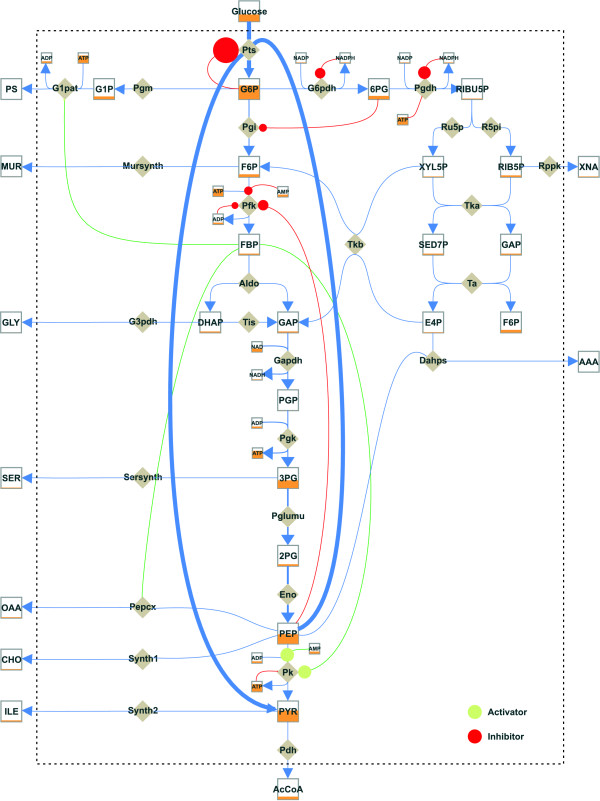
**Visualization of the dynamic metabolic model of *E. coli***. The respective dynamic data were generated using MMT2 [15]. The representation shows a metabolic snapshot of the current metabolite concentrations (box levels), metabolic fluxes (arrow widths) and effector influences (circle sizes) at a time point *t *= 0.1 s after the glucose pulse.

Figure 8 presents a visualization of the whole *E*. *coli *model designed with MetVis. It portrays the state of the animation after the substrate pulse of glucose is given (*t *= 0.1 s). In this case the animation underlies a global scaling, where the metabolite pools and flux values are divided by the overall time maximum of all concentration and flux values, respectively [16].

Thus, an empty metabolite pool indicates a concentration value of zero. Conversely, a full pool box indicates a global concentration maximum regarding all metabolite concentrations, e.g. the high intracellular concentration of G6P. This representation is very useful in comparing the global flux distribution in the network and the absolute changes in metabolite pool sizes. In this manner the identification of flux controlling or flux limiting steps in the network becomes possible.

##### Biological explanation

Referring to the visualized network the high glucose uptake flux via the Pts, which dominates the system directly after the glucose pulse, can be recognized. Obviously, at this early time point after the pulse the G6P converting enzymes, Pgi and G6pdh, are the most limiting steps for the further conversion of the glucose after uptake.

**Figure 9:**
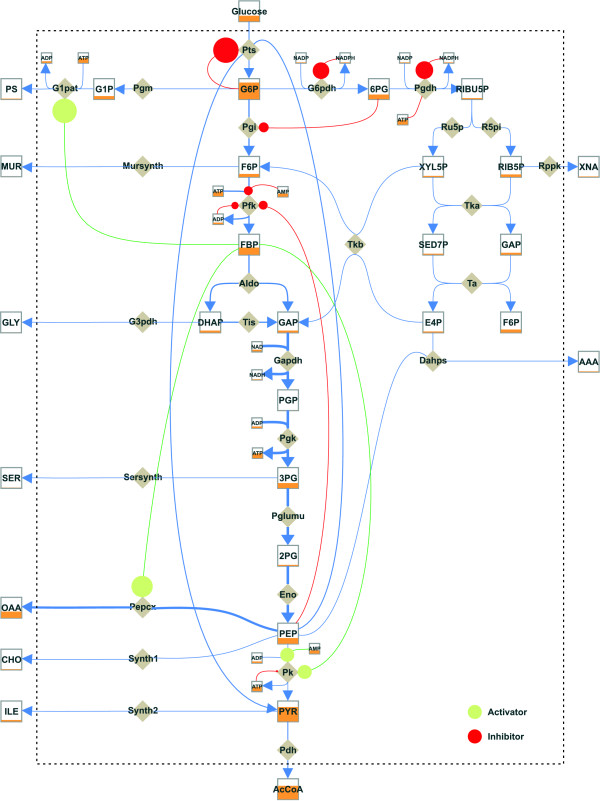
**Long-term pulse response of the *E. coli *network**. The visualization represents a metabolic snapshot at a time point *t *= 23.9 s after the glucose pulse.

As already mentioned, the Pts reaction is strongly inhibited by its product G6P and thus limits the glucose uptake in the following time course (cf. Figure 9). The activation effect that FBP exercises on G1pat, Pk and PEPCx increases substantially, thus favoring the production of OAA and increasing the consumption of PEP. The decrease in the concentration of the latter also decreases the inhibition effect on Pfk, leading to a general increase in OAA production.

The first two enzymes, G6pdh and Pgdh, catalyzing the entrance reaction into the pentose phosphate pathway are inhibited by NADPH, leading to a 50% decrease in their reaction rates. Despite the low concentration of NADPH in comparison with the second inhibitor ATP of the Pgdh enzyme, the inhibitory effect is dominated by NADPH. The reason for this effect is the very low affinity of Pgdh towards ATP (*K*_ATP _= 208 mM).

## Conclusion

A visualization approach has been presented that is suitable for an intuitive interpretation of simulation data under dynamic as well as steady-state conditions. Huge amounts of simulation data can be analyzed in a quick and comprehensive way. The visualization of regulatory interactions in a given metabolic network is based on a novel concept defining the RS of effectors regulating certain reaction steps. These RS values are measures for the strength of an up- or down-regulation of a reaction step compared with the completely non-inhibited or non-activated state, respectively. The concept of RS presented here is applicable to any mechanistic reaction kinetic formula.

So far this regulatory information has never been represented quantitatively in the available visualization tools. Hence, for the first time, by applying the proposed concept using the MetVis tool, a visualization of dynamic changes in metabolite pools, fluxes and RS data within the whole network structure becomes tractable. The incorporation of additional edges for effectors closes the information gap between obtained pool sizes and fluxes leading to the right interpretation of metabolic fluxes underlying a certain metabolic regulation.

One limitation of mechanistic enzyme descriptions is the large number of model parameters that have to be estimated according to given experimental data. This has led to the development of approximative kinetic rate equations based on the linlog or power law approach. Despite their broad applicability, one major drawback of these kinetic formats is their indeterminacy for concentration values of zero caused by the logarithmization of pool sizes. Clearly, to utilize the RS concept to the linlog and power law approaches, lower boundaries for the effector pools (***e***_min _> 0) also have to be defined.

By using the complex example system of the dynamic metabolic *E. coli *network, it has been shown that the extended time-resolved graphical network presentation provides a series of information about regulatory interaction within the biological system under investigation. Quantitative modeling has also become possible in the fields of transcriptomics and proteomics owing to the enormous increase in information available. Regulation at the genome level mainly takes place during the transcription of genes into mRNA, e.g. inhibition of RNA-polymerase through the binding of certain repressor proteins [18]. In contrast, the protein function is regulated by post-translational modifications such as phosphorylation [19].

The formulation of 'vertical' network models combining all levels of regulation (genome, transcriptome, proteome, metabolome) will help us to gain an insight into the complex cellular network in its entirety. In the case of mechanistic descriptions for the reactions taking place in the different 'omics' levels, the concept of RS can be applied to quantify and visualize all regulatory interactions.

## Abbreviations

ADP, adenosindiphosphate; AMP, adenosinmonophosphate; ATP, adenosintriphosphate; CSV, character separated values; *E. coli*, *Escherichia coli*; FBP, fructose-1,6-bisphosphate; G1pat, glucose-1-phosphate adenyltransferase; G6P, glucose-6-phosphate; G6pdh, glucose-6-phosphate dehydrogenase; GUI, graphical user interface; MetVis, Metabolic Visualizer; MMT2, Metabolic Modeling Tool 2; NAD, nicotinamide-adenindinucleotide; NADPH, nicotinamide-adenindinucleotide-phosphate; OAA, oxaloacetate; PEP, phosphoenolpyruvate; PEPCx, phosphoenolpyruvate carboxylase; Pfk, phosphofructokinase; Pgdh, 6-phosphogluconate dehydrogenase; Pgi, phosphoglucose isomerase; Pk, pyruvate kinase; Pts, phosphotransferase system; Pyr, pyruvate; RS, regulatory strength.

## Symbols used

*a*, *b*, rate constant factors; **e**_max_, vector of maximal effector concentrations; *K*_*i*_, affinity constant for a certain metabolite *i*; *r*_*j*_, reaction rate of enzyme *j*; *r*_max_, maximum reaction rate; *r*_min, **e**_, *r*_max, **e**_, minimal and maximal reachable flux for variable effector pools; *r*_0, **e**_, flux in the completely non-regulated state; S, A, I, substrate, activator and inhibitor pool; **s**, **e**, **a**, **i**, vector of substrate, effector, activator and inhibitor pools; *α*, *β*, affinity constant factors; ν˜ek
 MathType@MTEF@5@5@+=feaafiart1ev1aaatCvAUfKttLearuWrP9MDH5MBPbIqV92AaeXatLxBI9gBaebbnrfifHhDYfgasaacH8akY=wiFfYdH8Gipec8Eeeu0xXdbba9frFj0=OqFfea0dXdd9vqai=hGuQ8kuc9pgc9s8qqaq=dirpe0xb9q8qiLsFr0=vr0=vr0dc8meaabaqaciaacaGaaeqabaqabeGadaaakeaaiiGacuWF9oGBgaacamaaBaaaleaacqWGLbqzdaWgaaadbaGaem4AaSgabeaaaSqabaaaaa@3190@, scaled single effector influence; *ν*_res_, resulting RS; *ω*, scaling factor.

## Glossary

Effector: metabolite that modulates an enzyme-catalyzed reaction leading to an acceleration (activator) or deceleration (inhibitor) of the reaction rate.

Regulatory strength: measure for the strength of an up- or down-regulation of a reaction step compared with the completely non-inhibited or non-activated state.

Competitive inhibition: metabolites which are not substrates of the enzyme can bind to the active site and compete with the substrate.

Non-competitive inhibition: an inhibitor can bind to the enzyme substrate complex (ES) forming a complex (ESI) that reduces the amount of active enzyme.

Allosteric inhibition or activation: the enzyme has additional binding sites for specific inhibitors or activators which can change the conformation resulting in a change of the enzyme activity.

## Competing interests

The author(s) declares that there are no competing interests.

## Authors' contributions

SN developed the methods for the RS determination in metabolic networks. EQ developed the MetVis tool and helped to extend its functionality for the visualization of RS data. AW and WW conceived of the RS concept, participated in its elaboration and helped to draft the manuscript. All authors read and approved the manuscript.

## References

[B1] Kanehisa MG (2000). KEGG: Kyoto encyclopedia of genes and genomes. Nucleic Acids Res.

[B2] Krieger CJ, Zhang P, Mueller LA, Wang A, Paley S, Arnaud M, Pick J, Rhee SY, Karp PD (2004). MetaCyc: a multiorganism database of metabolic pathways and enzymes. Nucleic Acids Res.

[B3] Keseler IM, Collado-Vides J, Gama-Castro S, Ingraham J, Paley S, Paulsen IT, Peralta-Gil M, Karp PD (2005). EcoCyc: a comprehensive database resource for Escherichia coli. Nucleic Acids Res.

[B4] Schomburg I, Chang A, Ebeling C, Gremse M, Heldt C, Huhn G, Schomburg D (2004). BRENDA, the enzyme database: updates and major new developments. Nucleic Acids Res.

[B5] Klamt S, Stelling J, Ginkel M, Gilles E (2003). FluxAnalyzer: exploring structure, pathways, and flux distributions in metabolic networks on interactive flux maps. Bioinformatics.

[B6] Klamt S, Saez-Rodriguez J, Gilles ED (2007). Structural and functional analysis of cellular networks with CellNetAnalyzer. BMC Syst Biol.

[B7] Rost U, Kummer U (2004). Visualisation of biochemical network simulations with SimWiz. IEE Proc Syst Biol.

[B8] Qeli E, Wahl A, Degenring D, Wiechert W, Freisleben B (2003). MetVis: A tool for designing and animating metabolic networks. Proceedings of the 2003 European Simulation and Modelling Conference.

[B9] Junker BH, Klukas C, Schreiber F (2006). VANTED: A system for advanced data analysis and visualization in the context of biological networks. BMC Bioinformatics.

[B10] Brandes U, Dwyer T, Schreiber F (2003). Visualizing related metabolic pathways in two and a half dimensions. Proceedings of the 11th International Symposium on Graph Drawing.

[B11] Dwyer T, Rolletschek H, Schreiber F (2004). Representing experimental biological data in metabolic networks. Proceedings of the 2nd Asia Pacific Bioinformatics Conference.

[B12] Segel IH (1975). Enzyme Kinetics.

[B13] Rizzi M, Baltes M, Theobald U, Reuss M (1997). *In vivo *analysis of metabolic dynamics in *Sacharomyces cerevisiae*: II. Mathematical model. Biotechnol Bioeng.

[B14] Chassagnole C, Noisommit-Rizzi N, Schmid JW, Mauch K, Reuss M (2002). Dynamic modeling of the central carbon metabolism of *Escherichia coli*. Biotechnol Bioeng.

[B15] Haunschild MD, Freisleben B, Takors R, Wiechert W (2005). Investigating the dynamic behavior of biochemical networks using model families. Bioinformatics.

[B16] Oldiges M, Noack S, Wahl A, Qeli E (2006). From enzyme kinetics to metabolic network modeling–visualization tool for enhanced kinetic analysis of biochemical network models. Eng Life Sci.

[B17] Hofmann E, Kopperschlaeger G (1982). Phosphofructokinase from yeast. Meth Enzymol.

[B18] Wennerhold J, Krug A, Bott M (2005). The AraC-type regulator RipA represses aconitase and other iron proteins from Corynebacterium under iron limitation and is itself repressed by DtxR. J Biol Chem.

[B19] Niebisch A, Kabus A, Schultz C, Weil B, Bott M (2006). Corynebacterial protein kinase G controls 2-oxoglutarate dehydrogenase activity via the phosphorylation status of the OdhI protein. J Biol Chem.

